# Complete Circular Genome Sequence and Temperature Independent Adaptation to Anaerobiosis of *Listeria weihenstephanensis* DSM 24698

**DOI:** 10.3389/fmicb.2017.01672

**Published:** 2017-09-01

**Authors:** Elena Ferrari, Mathias C. Walter, Christopher Huptas, Siegfried Scherer, Stefanie Müller-Herbst

**Affiliations:** ^1^Chair of Microbial Ecology, Technische Universität München Freising, Germany; ^2^ZIEL—Institute for Food & Health, Technische Universität München Freising, Germany; ^3^Department of Genome-Oriented Bioinformatics, Technische Universität München Freising, Germany

**Keywords:** *Listeria weihenstephanensis*, complete circular genome sequence, adaptation to anaerobiosis, nitrate respiration, transcriptome, temperature

## Abstract

The aim of this study was to analyze the adaptation of the environmental *Listeria weihenstephanensis* DSM 24698 to anaerobiosis. The complete circular genome sequence of this species is reported and the adaptation of *L. weihenstephanensis* DSM 24698 to oxygen availability was investigated by global transcriptional analyses via RNAseq at 18 and 34°C. A list of operons was created based on the transcriptional data. Forty-two genes were upregulated anaerobically and 62 genes were downregulated anaerobically. The oxygen dependent gene expression of selected genes was further validated via qPCR. Many of the differentially regulated genes encode metabolic enzymes indicating broad metabolic adaptations with respect to oxygen availability. Genes showing the strongest oxygen-dependent adaption encoded nitrate (*narGHJI*) and nitrite (*nirBD*) reductases. Together with the observation that nitrate supported anaerobic growth, these data indicate that *L. weihenstephanensis* DSM 24698 performs anaerobic nitrate respiration. The wide overlap between the oxygen-dependent transcriptional regulation at 18 and 34°C suggest that temperature does not play a key role in the oxygen-dependent transcriptional regulation of *L. weihenstephanensis* DSM 24698.

## Introduction

*Listeria* are Gram-positive, facultative anaerobic, non-spore-forming, rod-shaped bacteria, 0.5 μm in width and 1.0–1.5 μm in length, with a low G+C content (McLauchlin and Rees, 2009), which can be isolated from a variety of environments, including soil, water, wastewater, sludge, feces, silage, food, and food processing environments (Paillard et al., 2005; Vilar et al., 2007; O'Connor et al., 2010; Linke et al., 2014). The genus *Listeria* currently contains 17 species which are, based on comparative genomics, divided in the *Listeria* sensu stricto and the *Listeria* sensu lato group (Chiara et al., 2015). The *Listeria* sensu stricto clade includes *Listeria monocytogenes* (Pirie, 1940), *Listeria innocua* (Seeliger, 1981), *Listeria seeligeri* (Rocourt and Grimont, 1983), *Listeria welshimeri* (Rocourt and Grimont, 1983), *Listeria ivanovii* (Seeliger et al., 1984), comprising the subspecies *L. ivanovii* subsp. *ivanovii* and *L. ivanovii* subsp. *londoniensis* (Boerlin et al., 1992) and *Listeria marthii* (Graves et al., 2010). The *Listeria* sensu lato group is further divided into three subclades (Chiara et al., 2015), for which recently even the implementation of three new genera was proposed (Orsi and Wiedmann, 2016). One group consists of only one species, *Listeria grayi* (Errebo Larsen and Seeliger, 1966), one includes *Listeria fleischmanii* (Bertsch et al., 2013), comprising the subspecies *L. fleischmannii* subsp. *coloradonensis* and *L. fleischmannii* subsp. *fleischmannii* (den Bakker et al., 2013), *Listeria floridensis*, and *Listeria aquatica* (den Bakker et al., 2014), and one is composed of *Listeria rocourtiae* (Leclercq et al., 2010), *L. weihenstephanensis* (Lang-Halter et al., 2013), *Listeria cornellensis, Listeria riparia, Listeria grandensis* (den Bakker et al., 2014), *Listeria newyorkensis*, and *Listeria booriae* (Weller et al., 2015).

The *Listeria* senu stricto group includes two pathogenic species, *L. monocytogenes* and *L. ivanovii. L. monocytogenes* infects both humans and animals and is known as an important opportunistic human foodborne pathogen, which causes, after ingestion of contaminated food, human listeriosis, a rare but severe disease (reviewed in Allerberger and Wagner, 2010). *L. ivanovii* is an animal pathogen (Gill et al., 1997; Chand and Sadana, 1999).

Whereas, especially the two pathogens *L. monocytogenes* and *L. ivanovii* which belong to the *Listeria* sensu stricto group are well-investigated, almost nothing is known about the members from the *Listeria* sensu lato group, which appear to be environmental species that have not yet been associated with human or animal diseases. In this study we investigated the adaptation of *L. weihenstephanensis* DSM 24698 to oxygen restriction. *L. weihenstephanensis* DSM 24698 was isolated from the water plant *Lemna trisulca* from a fresh water pond in Bavaria, Germany. The isolate is non-haemolytic and shows optimal growth at pH 7–8 and a temperature of 28–34°C (Lang-Halter et al., 2013).

The adaptation to oxygen restriction is of special interest, not only because the bacterium might encounter microaerophilic of anaerobic conditions in certain environmental niches, but also because the pathogens *L. monocytogenes* and *L. ivanovii* have to deal with oxygen restriction in the gastrointestinal tract of the host.

In this study, the adaptation of *L. weihenstephanensis* DSM 24698 to oxygen restriction was analyzed at two different temperatures. It has been reported earlier that in *L. monocytogenes* massive temperature dependent transcriptional changes can be observed. Best studied examples are the upregulated expression of flagellar genes at environmental temperature (Williams et al., 2005) and the increased expression of the classical *L. monocytogenes* virulence genes important for adhesion to and invasion into host cells and cell to cell spread at host temperature (reviewed in Vázquez-Boland et al., 2001). The temperature dependent regulation of many virulence genes is mediated by the transcriptional activator PrfA, whose activity is activated via a RNA thermosensor (Johansson et al., 2002). Furthermore, it has been shown recently that *L. monocytogenes* also regulates the expression of genes involved in central metabolic pathways like nitrogen metabolism in a temperature dependent manner (Kaspar et al., 2014), also suggesting a niche dependent metabolic adaptation.

To investigate whether the oxygen dependent metabolic adaptation in the environmental bacterium *L. weihenstephanensis* DSM 24698 is influenced by temperature, the transcriptional response to anaerobiosis was analyzed at 18°C, a potential environmental temperature, and 34°C.

## Materials and methods

### Strain and culture conditions

The strain used in this study was *L. weihenstephanensis* DSM 24689 (= WS 4560; Lang-Halter et al., 2013).

For cultivation on solid media, bacteria from the glycerol stock were cultivated on BHI agar and incubated for 1–2 days at 24°C and then stored at 4°C. For overnight liquid cultures, a single bacterial colony was inoculated in 50 ml BHI medium (Brain Heart Infusion, Merck, Darmstadt, Germany) overnight (17 h) at 24°C under continuous shaking (150 rpm). Overnight cultures were diluted 1:100 in fresh BHI medium in a 51-ml total volume for growth analysis. For aerobic growth the cultures were grown in 200-ml flasks, for anaerobic growth cultures were grown in 50-ml sealed CELLSTAR® tubes (Greiner Bio-One, Frickenhausen, Germany). Oxygen availability was analyzed in parallel cultures to which the redox indicator resazurin (0.0001% w/v, 7-Hydroxy-3H-phenoxazin-3-one-10-oxide; Sigma-Aldrich, Taufkirchen, Germany) was added. When oxygen in the medium became limited, resazurin changed color from blue to pink and further to colorless when the culture became anaerobic. All cultures were incubated at the specific experimental temperature under continuous shaking (150 rpm). The optical density at 600 nm (OD_600_) was measured using the spectrophotometer Lambda Bio+ (PerkinElmer, Rodgau, Germany) every hour until the stationary phase was reached. One additional measurement was taken after 24 h. Three biologically independent experiments were performed for each growth condition. The mean values of the growth analysis were used for the calculation of the doubling time and the maximum OD_600_ (OD_600 max_) with the GrowthRates v2.0 calculator (Hall et al., 2014).

### Small scale *in vitro* growth analyses

For *in vitro* growth analysis in a 200-μl micro-volume a Bioscreen C growth curve reader was used (Oy Growth Curves Ab Ltd., Helsinki, Finland). Overnight cultures were diluted 1:200 in a modified minimal medium supplied with 0 or 10 mM sodium nitrate (NaNO_3_). The composition of the medium with glutamine as nitrogen source was described previously (Kaspar et al., 2014). As carbon source 1 g L^−1^ D (+)-glucose monohydrate (Fluka, Darmstadt, Germany) was used. Furthermore, 5 g L^−1^ yeast extract (Oxoid, Wesel, Germany) were added. Cultures were incubated at 34°C with continuous medium shaking (shaking step 60). For anaerobic growth analyses, cultures were overlaid with 200 μl sterile paraffin oil (Roth, Karlsruhe, Germany). The OD_600_ of each well was automatically recorded every hour over a period of 24 h. Mean values and standard deviation were calculated from at least four independent biological experiments each including technical duplicates. A student's *t*-test (Two-Sample Assuming Unequal Variance, Microsoft Excel) was performed for statistical analysis.

### Genome sequencing using illumina

The *L. weihenstephanensis* DSM 24698 genome was sequenced on the MiSeq platform (Illumina, San Diego, USA) using v2 chemistry (400 cycles; Illumina, München, Germany). Genomic DNA was isolated from a 3-ml overnight culture grown at 30°C. The isolated DNA was diluted to 20 ng μl^−1^. Fifty-five microliters were transferred to a Snap Cap microTUBE (Covaris, Brighton, United Kingdom) and the DNA was fragmented in the Covaris sonnicator S220 (45 s, 175 watt, 10% duty factor, 200 cycles per burst). The sample was stored at −20°C until further processing. The library for sequencing was prepared with a modified version of the standard PCR-free TruSeq DNA sample preparation protocol (Huptas et al., 2016).

### Genome sequencing using PacBio

For DNA sequencing using the Pacific Biosciences PacBio RS II platform, an overnight culture was diluted 1:100 in BHI medium and grown aerobically at 18°C under constant shaking (150 rpm) to an OD_600_ = 0.86. The cells were pelleted by centrifugation (18°C, 8 min, 4,186 × g). The DNA isolation and sequencing run were performed by GATC Biotech (Konstanz, Germany).

### Genome assembly and annotation

The raw PacBio sequence data were assembled with the hierarchical genome-assembly process (Chin et al., 2013) v3 workflow of the SMRT Analysis software, v2.3. Then, Pilon (Walker et al., 2014) was used to improve the assembly further by incorporating the Illumina sequence data. The resulting contig was circularized manually. An unusual high coverage region was further analyzed. This region contained a prophage sequence. The attachment sites of this prophage were determined and the sequence was extracted from the chromosomal sequence and circularized. Afterwards, reads were mapped against the bacterial chromosomal integration site and the circularized phage chromosome to verify the correct breakpoint sequences.

Both sequences were submitted to GenBank and structurally annotated by the NCBI Prokaryotic Annotation Pipeline (PGAP; Tatusova et al., 2016). Functional annotation was performed by the RAST server (Overbeek et al., 2014) and the PEDANT system (Walter et al., 2009) to get additional functional annotation like Clusters of Orthologous Groups (COGs; Galperin et al., 2015) assignment or annotation of genes not characterized by PGAP.

The sequencing project was registered in the NCBI BioProject database with accession number PRJNA275474. Genomic raw sequence data were submitted to the sequence read archive (SRA) with accession number SRS845980. The GenBank accession number for the chromosome is CP011102 and for the phage genome CP011103.

### Transcriptional profiling

#### Cell harvesting and RNA extraction

*L. weihenstephanensis* DSM 24698 cultures (51 ml) were grown aerobically or anaerobically in BHI medium at the specific temperature to an OD_600_ = 0.85–0.95. The RNA was isolated following the protocol previously published (Müller-Herbst et al., 2014), but performing the disruption by bead-beating (FastPrep-24, MP Biomedicals, USA) four times for 45 s at 6.5 m s^−1^. After the first DNAse I (RQ1 RNase-Free DNase Promega, Mannheim, Germany) treatment, RNA was purified from proteins by chloroform extraction (100 μl). Total RNA from the aqueous phase was further purified using the RNeasy Mini Kit (Qiagen, Hilden, Germany) according to the manufacturer's RNA cleanup protocol. An additional on-column DNA digest (Qiagen RNase-free DNase, 2 U μl^−1^) was performed according to the manufacturer's instructions before RNA was eluted in nuclease-free water (30-μl final volume).

#### Library preparation and RNA sequencing

The RNAs were isolated as described above, but, after the second purification step with chloroform, only 60 μg RNA from the aqueous phase were further purified with the RNeasy Mini Kit following the RNA cleanup protocol including the optional on-column DNAse I treatment. Five micrograms of purified RNA was then mRNA-enriched by 16S and 23S rRNA depletion (MICROBExpress Kit, Ambion, Life Technologies, Darmstadt, Germany), following the manufacturers‘ instructions. The samples were then quantified using the 2.0 Qubit Fluorometer (Life Technologies, Darmstadt, Germany) and qualitatively analyzed by the Agilent 2100 Bioanalyzer (RNA 6000 Nano Kit; Agilent Technologies, Waldbronn, Germany). For library preparation, 300 ng of each total RNA were fragmented in 50 μl nuclease-free water (S220 sonicator, Covaris, 180 s, 175 watt, 10% duty factor and 200 cycles per burst) to obtain fragments with a normal distribution around 150 bp. The dephosphorylation of the RNA fragments was carried out using antarctic phosphatase (15 U), 1X antarctic phosphatase buffer (New England BioLabs, Frankfurt, Germany) with the addition of RNase inhibitor SUPERase In (65 units, Ambion, Life Technologies, Darmstadt, Germany) in a 65.5-μl final volume for 1 h at 37°C. Seven hundred microliters of QIAzol lysis reagent were added to the RNA sample. The RNAs were then purified with the miRNeasy Mini Kit following the protocol for purification of total RNA from animal cells omitting the cell homogenization steps (Qiagen) and eluted in a nuclease-free water (35.5-μl final volume). For phosphorylation, T4 Polynucleotide Kinase, 1X T4 Polynucleotide Kinase Buffer (New England BioLabs), ATP (1 mM, New England BioLabs), and RNase inhibitor SUPERase In (50 U) were added to the RNA (50-μl final volume) and the sample was incubated for 1 h at 37°C. Afterwards, RNA was purified again (miRNeasy Mini Kit) with a final elution in 30 μl nuclease free water. After further quality and quantity control by Bioanalyzer and Qubit 100 ng of the samples were finally concentrated to 20 ng/μl by a Concentrator 5301 (Eppendorf, Hamburg, Germany). Ligation of 3′- and 5′-adapters, reverse transcription and PCR amplification were performed following the instructions from the TruSeq Small RNA Sample Preparation Guide (Illumina, February 2013). The cDNAs derived from the aerobic and anaerobic culture were mixed, before the size separation of the amplified cDNA fragments on a ready to use 6% Novex TBE polyacrylamide gel (Life Technologies, Darmstadt, Germany) was performed. The fragments were size-selected between 200 and 350 bp and purified by filtration (0.22 μm Spin-X filter, Corning, USA) and precipitation. The indexed libraries were diluted to a 2-nM concentration in Buffer EB (Qiagen). For denaturation, 10 μl of 0.1 M NaOH (1 M NaOH [pH 13.5] was diluted with buffer EB to a 0.1 M NaOH solution) were added to 10 μl of the 2-nM library and the sample was incubated for 5 min a RT. Finally, the library was diluted with pre-chilled HT1 buffer (Illumina) to a concentration of 10 pM in 0.5 mM NaOH. Finally, the libraries were sequenced on the MiSeq sequencer (Illumina) using a MiSeq Reagent Kit v2 (50 cycles, Illumina) resulting in 50 bp single-end reads.

### Bioinformatics

The Illumina RNA-Seq reads were mapped to the chromosomal sequence of *L. weihenstephanensis* DSM 24698 using Bowtie for Illumina implemented in Galaxy (Blankenberg et al., 2010; Goecks et al., 2010) with commonly used settings (seed length 28 nt, maximum number of mismatches in the seed is 2). Data processing steps to receive SAM and BAM files were performed as described previously (Landstorfer et al., 2014). The number of reads mapping on each gene were visualized and calculated in Artemis v. 15.0.0 (Rutherford et al., 2000; Carver et al., 2012), based on the GenBank file of the *L. weihenstephanensis* DSM 24698 reference genome. The number of reads for all conditions were normalized to the total number of reads of the smallest library (*L. weihenstephanensis* DSM 24698 18°C anaerobic) for a more accurate comparison between temperatures.

A second transcript analysis tool (Rockhopper, McClure et al., 2013) was used for verification and to determine non-coding RNAs (ncRNAs) as well as to infer and annotate operon structures (Fortino et al., 2014).

### Differential expression analysis for the RNA-seq data

Differential gene expression was analyzed according to Mühlig et al. (2014), using the Bioconductor package edgeR (Robinson et al., 2010). Genes with ≤1 cpm (counts per million) in total under both the aerobic and anaerobic condition were filtered and not used in the analysis. The transcription of genes under aerobic conditions served as reference. Upregulated genes are expected to show a stronger transcription anaerobically, while downregulated genes are expected to be less transcribed upon oxygen-limitation. Genes showing a log_2_fold change in relative transcription (log_2_FC) ≥ 1 or ≤ −1, a false discovery rate (FDR) < 0.3 and a BH-corrected *p* < 0.05 were considered as being significantly up- or downregulated. The data discussed in this paper have been deposited in NCBI′s Gene Expression Omnibus (Edgar et al., 2002) and are accessible through GEO Series accession number GSE94124 (https://www.ncbi.nlm.nih.gov/geo/query/acc.cgi?acc=GSE94124).

### Quantitative real time PCR (qPCR)

To validate the RNA-Seq data, qPCR analyses were performed as previously described (Müller-Herbst et al., 2014). The qPCR reactions were carried out in duplicates for three biologically independent repetitions, which were also independent of the samples used for RNA-Seq, in an ICycler (Bio-Rad Laboratories GmbH, München, Germany) or in a SmartCycler® System (Cepheid, CA, USA). Oligonucleotides used for qPCR analyses are summarized in Table 1. Data were analyzed using the 2^−ΔΔCt^ method (Schmittgen and Livak, 2008). Transcription of the housekeeping gene encoding ClpX, the ATP-binding subunit of ATP-dependent Clp protease, was used for normalization.

**Table 1:** Oligonucleotides used in this study.

**Gene**	**Name**	**Sequence 5′–3′**
*UE46_00145, cat*	UE46_00145_qRT_F	gaggctacgacgatgattac
	UE46_00145_qRT_R	aacgcattttgatgtaaacc
*UE46_00920, nrdD*	UE46_00920_qRT_F	cgaaactggtattttcgatt
	UE46_00920_qRT_R	taattcagcacatctgggta
*UE46_01305, nirD*	UE46_01305_qRT_F	cataaaaacggtccactagc
	UE46_01305_qRT_R	cagtttcatacgttttgacg
*UE46_01505, clpX*	UE46_01505_qRT_F	ggctgattttgatgtagagc
	UE46_01505_qRT_R	tgagtaaggcttgttgcac
*UE46_03380*	UE46_03380_qRT_F	gactgcaaaaggaagatgag
	UE46_03380_qRT_R	caatataagctcgctgctg
*UE46_05890*	UE46_05890_qRT_F	agttattccgctacacatcg
	UE46_05890_qRT_R	cacattaggctgagaagagg
*UE46_08310, pflB*	UE46_08310_qRT_F	atgggtaacagaatcaatcg
	UE46_08310_qRT_R	agtcgaccaaagtacggtta
*UE46_08610, gcvPA*	UE46_08610_qRT_F	acaagaaattgagccgttag
	UE46_08610_qRT_R	cttgggaatctcctacaaca
*UE46_10735, gltB*	UE46_10735_qRT_F	cgattgacgtctttacgaat
	UE46_10735_qRT_R	caattttcgacatcaccttc
*UE46_11895, pdhB*	UE46_11895_qRT_F	aaggtcttttgatctcagca
	UE46_11895_qRT_R	ctttaccgatttcgattgtg
*UE46_11930, moaC*	UE46_11930_qRT_F	tattaaagaagggcaaatcg
	UE46_11930_qRT_R	actttcgtcgtcataattgg
*UE46_11975, narH*	UE46_11975_qRT_F	caactggaaaacgaataagg
	UE46_11975_qRT_R	cataaagcatcacaccaaga
*UE46_14170*	UE46_14170_qRT_F	attgcaccgaatatcttcac
	UE46_14170_qRT_R	aaatgctttcgtcaaactgt
*UE46_14320*	UE46_14320_qRT_F	cgaataaaatcgacttcgtg
	UE46_14320_qRT_R	cgaccgacttaatatccgta

## Results

### Complete genome sequence of *L. weihenstephanensis* DSM 24698

A key prerequisite for the analyses of the transcriptional adaption of *L. weihenstephanensis* DSM 24698 to the oxygen availability via RNA NGS was a high quality genome sequence. Up till now, only a draft genome sequence (den Bakker et al., 2014), finally resulting in 71 contigs (GenBank accession number AODJ00000000.1) was available. In this study, the complete genome of *L. weihenstephanensis* DSM 24698 was analyzed via in-house genome sequencing (MiSeq) and additional automated sequencing (PacBio RS II). The assembly of 1,789,120 high quality reads with a mean length of 189 bp from the MiSeq sequencing reaction resulted in 23 contigs with a total assembly size of 3,380,080 bp. The assembly of the PacBio RS II sequencing reaction of 78,900 high quality reads with a mean read length of 6,091 bp resulted, after incorporation of the MiSeq data, in one single circular chromosome with a ~50- to 180-fold coverage (Figure S1).

Worth mentioning is that after the assembly a ~40 kb region with an unusual high coverage pattern followed by a region with below average coverage was observed (Figure S1). This region was further analyzed using PHAST (Zhou et al., 2011) and revealed to encode phage genes. The observed abnormal coverage pattern would be explained if it is assumed that in the culture used for sequencing a lysogenic phage switched to the lytic cycle. The circularized phage chromosome of *L. weihenstephanensis* DSM 24698 phage 01 (LWP01) has a size of 41,687 bp and contains 57 coding regions. Blast analysis revealed that the protein sequence of the phage DNA polymerase (*UE_5225*) and a putative phage tail tape measure protein (*UE_5365*) matched specifically with proteins encoded in the genome of *L. monocytogenes* and *L. ivanovii* strains with a 79–82% and 79–81% identity, indicating its putative relationship to other *Listeria* phages.

The complete genome sequence of *L. weihenstephanensis* DSM 24698 including the prophage sequence has a size of 3,406,292 bp and a GC-content of 41.51%. Three thousand two hundred twenty-nine genes were identified via the automated annotation from NCBI, including 3,008 coding genes, 131 pseudogenes, 19 rRNAs (5S, 16S, 23S), 70 tRNAs and 1 non-coding RNA. One thousand nine hundred thirty-five genes were assigned to one or more COG categories (Table S1). Based on transcriptional data (see below) 647 putative operons which comprised 1,951 genes (Table S2) and 210 putative non-coding RNAs, among them 10 long non-coding RNAs (>200 bp; Table S3), were identified.

Interestingly, the chromosome of *L. weihenstephanensis* DSM 24698 harbors genes encoding a nitrate (*UE46_11980-UE46_11975*-*UE46_11970-UE46_11965, narGHJI*) and a nitrite (*UE46_01300*-*UE46_01305, nirBD*) reductase. These genes are absent in *L. monocytogenes* and all other members of the *Listeria* sensu stricto phylogenetic clade, but are present, at least partially, in all recently described species of the *Listeria* sensu lato clade, except for *L. floridensis*.

### Aerobic and anaerobic growth of *L. weihenstephanensis* DSM 24698

**Figure 1:**
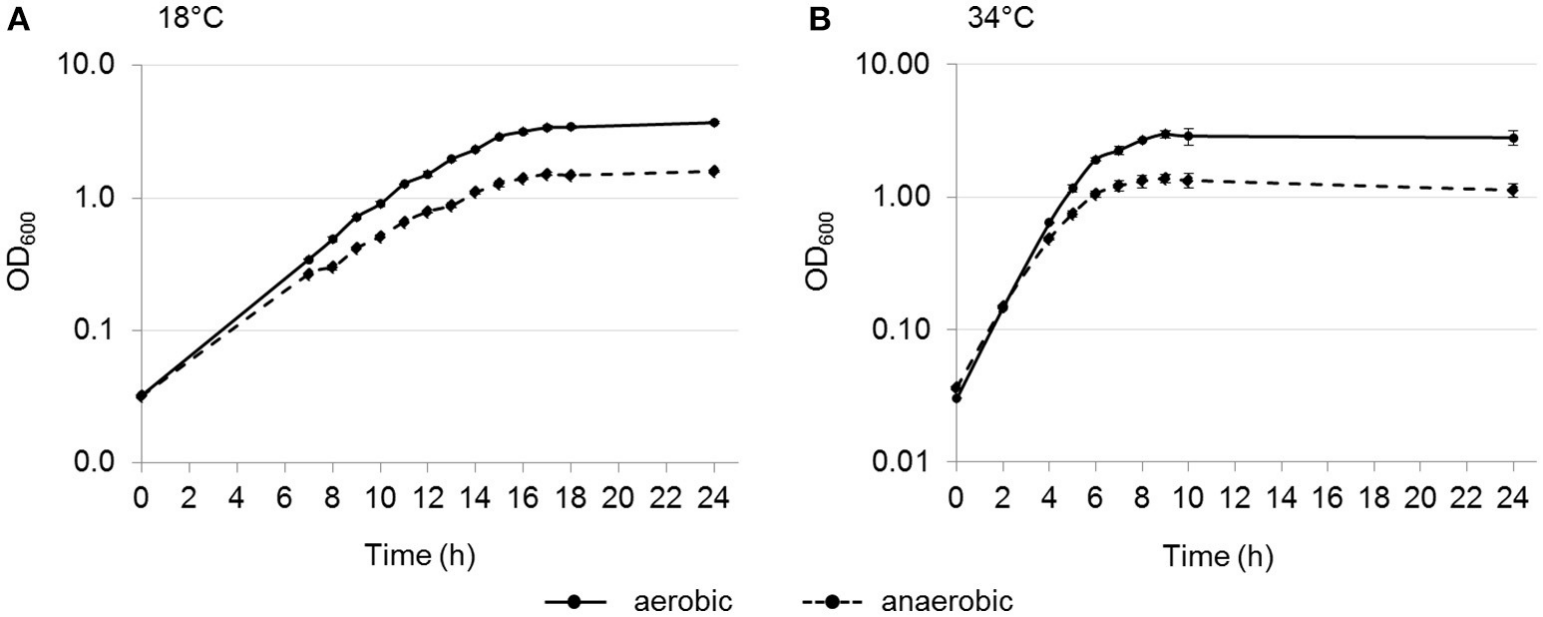
Oxygen-dependent growth of *L. weihenstephanensis* DSM 24698. Growth curves for *L. weihenstephanensis* DSM 24698 grown at 18°C **(A)** and 34°C **(B)** aerobically (solid line) and anaerobically (dotted line) are shown. OD_600_ was measured every hour. All data points represent means ± standard error from three independent biological replicates.

It has already been described that *L. weihenstephanensis* DSM 24698 is able to grow anaerobically on solid medium (Lang-Halter et al., 2013). To further investigate the oxygen dependent growth of *L. weihenstephanensis* DSM 24698, the bacterium was grown aerobically and anaerobically at 18 and 34°C in liquid culture. *L. weihenstephanensis* DSM 24698 grew aerobically and anaerobically at both temperatures (Figure 1). At both temperatures the OD_600 max_ was higher for aerobic compared to anaerobic growth (18°C: aerobic OD_600 max_ = 3.697, anaerobic OD_600 max_ = 1.590; 34°C: aerobic OD_600 max_ = 2.980, anaerobic OD_600 max_ = 1.380). Although, the OD_600 max_ for aerobic, respectively anaerobic growth was higher at 18°C than at 34°C, the doubling time at 18°C was higher than at 34°C (aerobic: doubling time at 18°C = 123.7 ± 1.8 min, doubling time at 34°C = 59.3 ± 2.6 min; anaerobic: doubling time at 18°C = 148.3 ± 5.7 min, doubling time at 34°C = 73.1 ± 5.0 min). At both temperatures the doubling time for anaerobic growth was higher than that for anaerobic growth.

### Oxygen dependent transcriptional adaptation of *L. weihenstephanensis* DSM 24698 at 18 and 34°C

To analyze the oxygen dependent transcription of *L. weihenstephanensis* DSM 24698, bacteria were cultivated aerobically and anaerobically at 18 and 34°C to an OD_600_ of 0.85–0.95 before cells were harvested for RNA isolation. At this OD_600_ resazurin controls indicated high or low oxygen availability.

**Figure 2:**
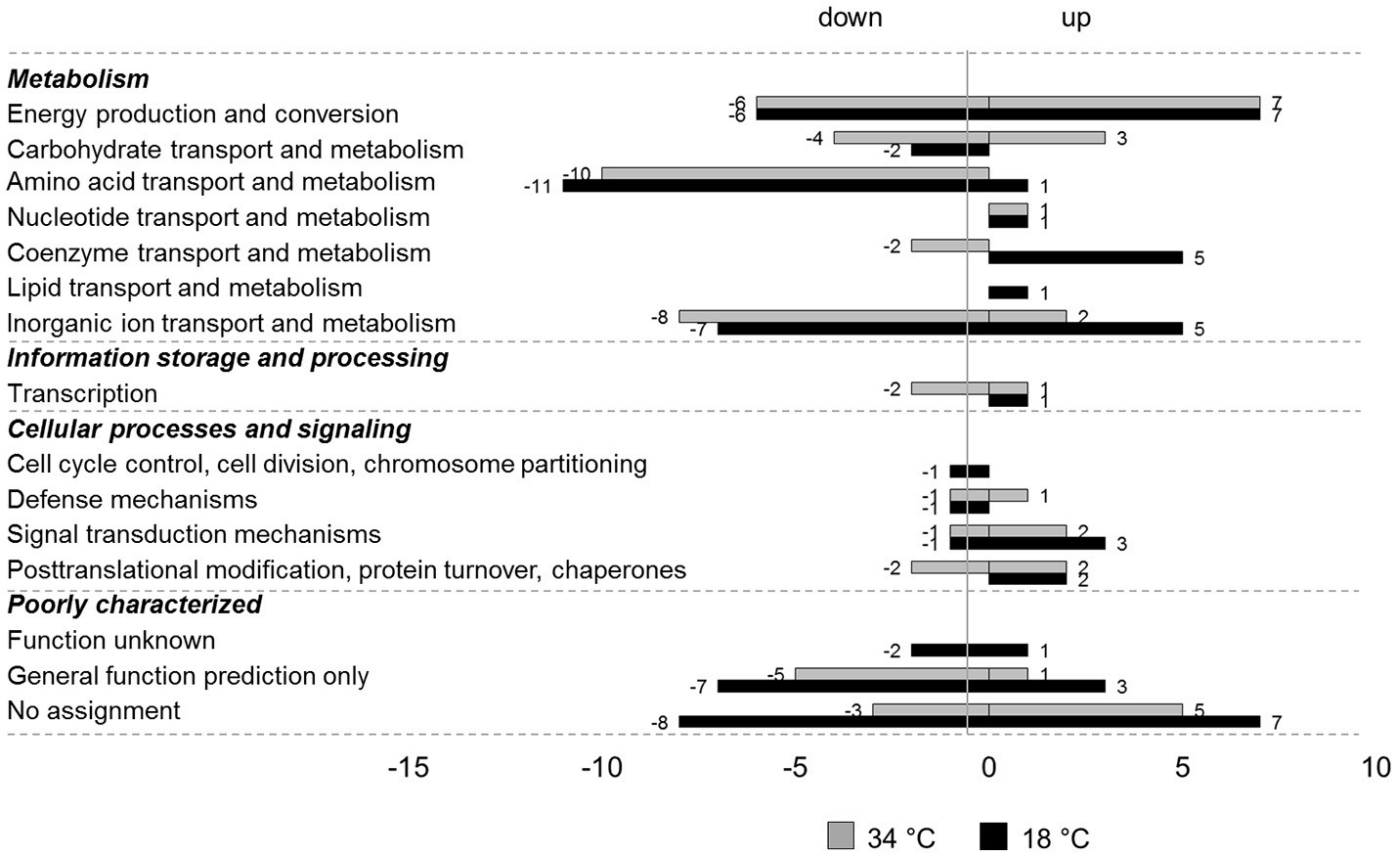
COG classification of genes regulated by oxygen at 18 and 34°C in *L. weihenstephanensis* DSM 24698. Genes up- or downregulated anaerobically at 18°C (black bars) or 34°C (gray bars) in *L. weihenstephanensis* DSM 24698 were grouped according to the NCBI COGs. Bars represent the number of genes up- or down-regulated for each category. Since one gene can belong to several COG categories the total number of regulated genes given in this figure is higher than the absolute number of regulated genes.

In total, 104 genes showed an oxygen dependent change in transcription. The anaerobically up- and down-regulated genes are listed in the Supplementary Material [Table S4a (upregulated genes) and Table S4b (downregulated genes)]. Forty-two genes were upregulated anaerobically and 62 genes were downregulated anaerobically. Most of the genes that were identified as being regulated depending on the oxygen availability only at one of the investigated temperatures showed the same tendency of regulation also at the other temperature, albeit not fulfilling all the filtering criteria applied during data analysis. For a better overview, the regulated genes were functionally classified according to their COGs (Figure 2).

Most of the anaerobically downregulated genes encode proteins involved in metabolism, in particular in energy production and conversion, amino acid transport and metabolism and inorganic ion processes. Some genes were involved in transcription and in cellular processes and signaling and many were poorly characterized with an unknown or only predicted function or not assigned to any COG category. Genes exhibiting a high negative log_2_FC at both the temperatures encoded for example catalase (*UE46_00145, cat*), pyruvate dehydrogenase (*UE46_11900-UE46_11895-UE46_11890-UE46_11885, pdhABCD*) and glutamate synthase (*UE46_10735 and UE46_10730, gltBD*; Table S4b).

Also, for the upregulated genes the majority of the regulated genes encoded proteins involved in metabolic pathways in particular in energy, carbohydrate, coenzyme, and inorganic ion processes. Again, many regulated genes were poorly characterized. A small number of the anaerobically upregulated genes were involved in transcription and in cellular processes and signaling. Among the anaerobically upregulated genes the genes showing the highest log_2_FC at both temperatures encoded nitrate (*narGHJI*) and nitrite (*nirBD*) reductase.

### Validation of RNA sequencing

**Figure 3:**
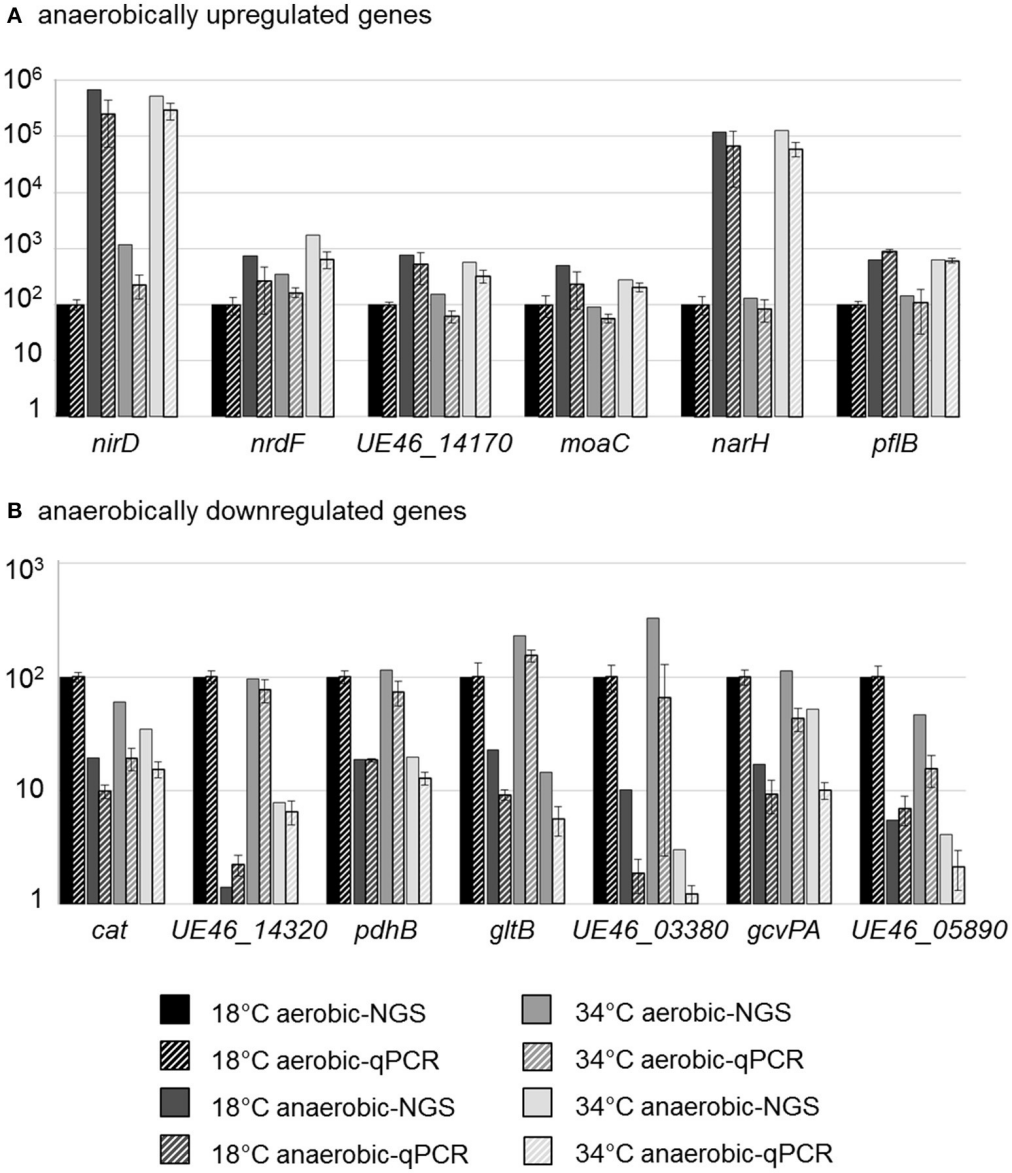
qPCR validation of RNA-NGS data for differently expressed genes. Bars represent the transcriptional level of selected upregulated **(A)** or downregulated **(B)** genes analyzed by NGS (solid bars) or qPCR (striped bars). The depicted data show the relative transcription in % compared to the reference condition (18°C aerobic) which was set to 100%. Bars for qPCR data represent mean values and standard errors from three biologically independent experiments, including technical duplicates.

To validate the transcriptional data, qPCR analyses of selected genes were performed. Among the anaerobically upregulated genes, the genes encoding subunits of the nitrate and nitrite reductases (*narH, nirD*), the molybdenum cofactor biosynthesis protein MoaC (*UE46_11930, moaC*), the catalytic subunit of class III anaerobic ribonucleoside-triphosphate reductase (*UE46_00920, nrdD*), a protein annotated as hypothetical by NCBI (NCBI_PGAP) but annotated as nitric oxide dioxygenase by RAST (*UE46_14170*), and a pyruvate formate lyase (formate acetyltransferase, *UE46_08310, pflB*) were selected (Figure 3A).

Among the anaerobically downregulated genes, those encoding subunits of pyruvate dehydrogenase (*pdhB*), of glutamate synthase (*gltB*), of glycine dehydrogenase, which is part of the glycine cleavage system in *L. monocytogenes* (Mujahid et al., 2013; *UE46_08610, gcvPA*), catalase (*cat*), an ATP binding protein of a putative iron ABC transporter (*UE46_03380*), a putative L-cystine-binding protein (*UE46_14320*), and a hypothetical protein (*UE46_05890*) were investigated (Figure 3B).

Results from qPCR analysis confirmed the data from the RNA-Seq analysis, which therefore can be regarded as veritable.

### Nitrate supported anaerobic growth of *L. weihenstephanensis* DSM 24698

The genome analysis revealed that nitrate and nitrite reductases are present in *L. weihenstephanensis* DSM 24698. The genes *narGHJI* and *nirBD* showed the strongest transcriptional induction during anaerobic growth, leading to the hypothesis that under anaerobic growth conditions *L. weihenstephanensis* DSM 24698 might switch from aerobic respiration to nitrate respiration.

**Figure 4:**
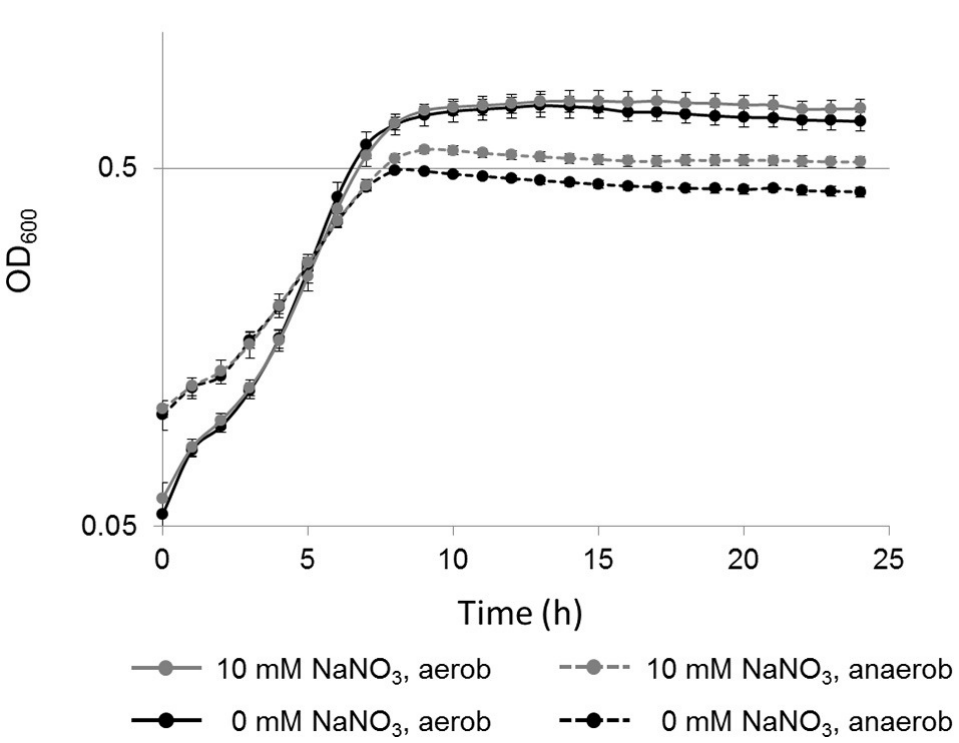
Nitrate-dependent growth of *L. weihenstephanensis* DSM 24698. Growth curves (y-axis logarithmic scale) for *L. weihenstephanensis* DSM 24698 grown at 34°C in a modified minimal medium aerobically (solid lines) and anaerobically (dotted lines), in the presence (gray lines) or absence (black lines) of nitrate (10 vs. 0 mM NaNO_3_) are shown. OD_600_ was measured every hour. All data points represent means ± standard error from seven independent biological replicates. Statistically significant differences in growth between cultures supplemented with NaNO_3_and those without NaNO_3_ were observed during anaerobic (student's *t*-test, *p* < 0.05 for time point 8 h, *p* < 0.005 for time points 9–24 h), but not during aerobic growth.

To test the hypothesis that the presence of nitrate could support the anaerobic growth of *L. weihenstephanensis* DSM 24698, growth analyses were performed. Growth was analyzed (Bioscreen C) at 34°C in the presence and absence of sodium nitrate (10 vs. 0 mM) under both aerobic and anaerobic conditions. First experiments in BHI medium did not reveal a significant growth induction in the presence of sodium nitrate (data not shown). However, the same experiments performed in a modified minimal medium (Figure 4) showed that anaerobically the presence of sodium nitrate led to a statistically significant increase of growth which was characterized by gaining a higher OD_600_ before reaching the stationary phase. Also, in the aerobic culture a slight growth induction in the presence of sodium nitrate was observed, albeit this increase was not statistically significant. It might well be that that at high cell densities a low oxygen tension in the medium results in a switch to nitrate respiration in the “aerobic” culture.

## Discussion

### Genome sequence of *L. weihenstephanensis* DSM 24698

In this study the genome sequence of *L. weihenstephanensis* DSM 24698 was revealed, being the first complete circularized chromosome of a member of the *Listeria* sensu lato phylogenetic group (Chiara et al., 2015). With a size of 3.4 Mbp the genome of *L. weihenstephanensis* DSM 24698 is larger than the genome of the members of the *Listeria* sensu stricto group with a size of ~2.8–3.0 Mbp (Glaser et al., 2001; Hain et al., 2006; den Bakker et al., 2010; Steinweg et al., 2010; Buchrieser et al., 2011), but comparable to the genome size of *L. rocourtiae, L. cornellensis, L. riparia, L. grandensis, L. newyorkensis*, and *L. booriae* (3.2–3.5 Mbp; den Bakker et al., 2014; Weller et al., 2015), which belong to the same *Listeria* sensu lato phylogenetic subclade as *L. weihenstephanasis* (Chiara et al., 2015). The main virulence genes from the human pathogen *L. monocytogenes* which allow attachment to and invasion into host cells and cell to cell spread are missing in *L. weihenstephanensis* DSM 24698 as well as in all other members of the *Listeria* sensu lato group, which readily explains why none of these recently described species has been associated with human or animal disease.

Genes present in *L. weihenstephanensis* DSM 24698 and at least partially in all the other members of the *Listeria* sensu lato phylogenetic clade, except for *L. floridensis*, are the genes encoding nitrate and nitrite reductases. This goes in line with the observation that all the members of the *Listeria* sensu lato group except for *L. floridensis* have the capability to reduce nitrate (den Bakker et al., 2014).

### Anaerobic nitrate and nitrite reduction in *L. weihenstephanensis* DSM 24698

The genes encoding nitrate reductase (*narGHJI*) were induced during anaerobic growth of *L. weihenstephanensis* DSM 24698 in BHI medium which did not contain supplementary nitrate. This indicates an oxygen-dependent transcriptional regulation of the *nar*-genes, which has also been described for other bacteria like *Staphylococcus aureus* (Fuchs et al., 2007) and *Bacillus subtilis* (Ye et al., 2000). Interestingly, the addition of sodium nitrate did not support anaerobic growth of *L. weihenstephanensis* DSM 24698 in BHI medium, but in a modified minimal medium, suggesting that nitrate respiration causes a growth advantage only under nutrient limited conditions. Preliminary results indeed indicate an accumulation of nitrite in the supernatant of anaerobically grown *L. weihenstephanensis* DSM 24698 cultures in modified minimal medium to which sodium nitrate was added (Table S5). Interestingly, the amount of glucose that was added as carbon source to the minimal medium seamed to influence the reduction of nitrate to nitrite. The less glucose was present in the medium, the more nitrite was detected in the culture supernatant. It is therefore tempting to speculate, that in BHI, which contains glucose in a concentration of 2 g L^−1^, the presence of glucose inhibits nitrate reduction. This would explain the observation that in BHI the supplementation of nitrate did not improve anaerobic growth. Such an inhibitory effect of glucose on nitrate respiration has also been described for other bacteria like *Lactobabacillus plantarum* (Brooijmans et al., 2009).

Besides genes encoding nitrate reductase, a gene cluster, which is located next to the nitrate reductase operon and which encodes genes involved in the biosynthesis of the molybdenum cofactor (*UE46_11960*-*UE46_11920*) was anaerobically stronger transcribed than aerobically. The molybdenum cofactor is essential for the functionality of nitrate reductases (reviewed in Coelho and Romão, 2015). Nitrate respiration has neither been described yet for any *Listeria* spp. nor for any member of the genus *Brochothrix*, the other genus in the family *Listeriaceae*. However, other members of the order *Bacillales* like *B. subtilis* (Hoffmann et al., 1998) and *Staphylococcus carnosus* (Neubauer and Götz, 1996) are able to perform anaerobic nitrate respiration.

Both, *B. subtilis* and *S. carnosus* further reduce nitrite to ammonia (Neubauer and Götz, 1996; Hoffmann et al., 1998). Our data indicate that nitrite reduction to ammonia might also occur in *L. weihenstephanensis* DSM 24698. We observed a strong upregulation of genes encoding nitrite reductase (*nirBD*) under anaerobic conditions. A reduction of nitrite to ammonia under anaerobic conditions would supply ammonia which could be used for anabolic processes.

It has been shown recently that ammonium is a better nitrogen source for *L. monocytogenes* than glutamine (Kaspar et al., 2014). Under low ammonium concentrations like e.g., in the host cell cytosol, transcription of genes encoding glutamine synthase (*glnA*), and glutamate synthase (*gltAB*) was induced in *L. monocytogenes*. In this study, a stronger transcription of *gltAB* was observed during aerobic growth, a condition under which ammonium could not be generated from nitrate via nitrite by anaerobic respiration. Furthermore, the stronger transcription of genes encoding a putative glutamine ABC transporter (UE46_13335 and UE46_13340 [*glnQ*]) and an ammonia channel protein (UE46_09395) aerobically might reflect a lower nitrogen source supply under aerobic conditions.

Worth mentioning is also the anaerobic upregulation of a gene encoding a putative nitric oxide dioxygenase (*UE46_14170*). This enzyme might be involved in the detoxification of nitric oxide, which can be produced in the presence of nitrite via Nar-type nitrate reductases (Vine et al., 2011; Rowley et al., 2012).

### Common features and differences in the oxygen dependent adaptation of *L. weihenstephanensis* DSM 24698 and *L. monocytogenes*

All these adaptations of *L. weihenstephanensis* DSM 24698 to changes in oxygen supply described so far were not reported for *L. monocytogenes*, which might be due to the lack of nitrate and nitrite reductases in the human pathogen. However, some similarities between the oxygen dependent adaptations of these two species were also observed. Anaerobically, transcription of genes encoding an anaerobic ribonucleosid-triphosphat reductase system (*nrdDG*) was induced both in *L. weihenstephanensis* and in *L. monocytogenes* (Müller-Herbst et al., 2014).

In both organisms the gene encoding catalase (*cat*) was stronger transcribed aerobically. This protein is involved in the detoxification of hydrogen peroxide, a toxic byproduct of aerobic respiration. Furthermore, in both species there was a stronger aerobic transcription of genes encoding proteins of the glycine cleavage system [*gcvT-gcvPA-gcvPB*; *lmo1348-lmo1350* in *L. monocytogenes* (Mujahid et al., 2013; Müller-Herbst et al., 2014), *UE46_08615-UE46_08610-UE46_08615* in *L. weihenstephanensis* DSM 24698]. The net reaction catalyzed by the glycine cleavage system is the reversible conversion of Glycine + H_4_folate + NAD^+^ to 5,10-methylene-H_4_folate + CO_2_ + NH_3_ + NADH + H^+^ (reviewed in Kikuchi et al., 2008). Together with a serine hydroxymethyltransferase (*UE46_02440*), which was also stronger transcribed aerobically in *L. weihenstephanensis* DSM 24698, the overall reaction catalyzes the conversion of 2 glycine + NAD^+^ + H_2_O to serine + CO_2_ + NH_3_ + NADH + H^+^ (reviewed in Kikuchi et al., 2008). In both reactions NH_3_ and NADH/H^+^ could arise. Because of the need for balancing the NADH/H^+^ to NAD^+^ ratio to assure the catabolic capacity of the organism, it makes perfect sense for *L. monocytogenes* to reduce NADH/H^+^ generating reactions anaerobically, when NADH/H^+^ can only be converted to NAD^+^ via fermentative pathways and not via the respiratory chain. In *L. weihenstephanensis* DSM 24698, in which a respiratory chain is supposed to be active during anaerobic nitrate respiration, the main function of these systems might be the provision of NH_3_ as nitrogen source under aerobic growth conditions.

Also the genes encoding another NADH/H+ producing enzyme complex, the pyruvate dehydrogenase, were anaerobically downregulated in both *L. weihenstephanensis* and *L. monocytogenes*. Conversion of pyruvate to acetyl-CoA via pyruvate dehydrogenase is the major pathway to remove pyruvate from the metabolic pool during aerobic growth. However, also anaerobically pyruvate must be removed to allow carbon catabolism to continue. In *L. monocytogenes* pyruvate is mainly converted to lactate via lactate dehydrogenase anaerobically, thereby recycling NADH/H^+^ to NAD (Romick et al., 1996). In *L. weihenstephanensis* DSM 24698 the physiological role of the anaerobic downregulation of pyruvate dehydrogenase remains to be elucidated. However, downregulation of pyruvate dehydrogenase genes during nitrate respiration has also been described for *B. subtilis* (Ye et al., 2000).

In *L. weihenstephanensis* DSM 24698 an anaerobic upregulation of a gene cluster encoding a pyruvate formate lyase-activating protein (*pflC*) and a formate acetyltransferase (*pflB*) was observed, which together are responsible for the conversion of pyruvate to acetyl-CoA and formate. Formate can be used as electron donor in the nitrate respiratory chain (reviewed in Unden and Bongaerts, 1997) and could thereby compensate the lower production of NADH/H^+^.

In general, our data indicated that, albeit there are some similarities between the adaptations to oxygen concentration in *L. monocytogenes* and *L. weihenstephanensis* DSM 24698, the general adaptation to anaerobiosis differs in these two closely related species. In both organisms a gene encoding the subunit E1 of 2-oxoglutarate dehydrogenase is missing (Glaser et al., 2001, this study) causing a split of the citric acid cycle into an oxidative and a reductive branch, which has been shown via elegant physiological experiments for *L. monocytogenes* (Trivett and Meyer, 1971; Eisenreich et al., 2006). An incomplete citric acid cycle further implicates that pyruvate can't be oxidized completely to carbon dioxide. Even aerobically, fermentative pathways have to be used simultaneously to aerobic respiration, not to generate energy, but to get rid of pyruvate, the accumulation of which would inhibit glucose catabolism. Anaerobically, *L. monocytogenes* shifts its metabolism to fermentation only to ensure its energy production. *L. weihenstephanensis* DSM 24698 on the other site has the capacity to perform nitrate respiration for energy production during anaerobic growth. Nevertheless, both aerobic respiration and anaerobic nitrate respiration has to be accompanied by fermentative pathways to get rid of pyruvate.

### Temperature-independent adaptation to oxygen availability

For *L. monocytogenes* it has been shown previously that temperature has a massive impact on transcriptional regulation. Best studied examples are the temperature dependent regulation of virulence genes via PrfA (Leimeister-Wächter et al., 1992; Johansson et al., 2002), of flagella genes (Williams et al., 2005) and metabolic adaptations, e.g., temperature dependent expression of genes encoding enzymes involved in nitrogen metabolism (Kaspar et al., 2014). These temperature-dependent adaptations are thought to allow a perfect adaptation to the respective ecological niche as saprophyte in the environment or as an intracellular pathogen in the human host (reviewed in Freitag et al., 2009). The study of the oxygen dependent adaptation of *L. weihenstephanensis* DSM 24698 at 18 and 34°C, performed in this study, did not reveal a massive impact of the temperature on transcription, at least not on the oxygen dependent adaptation. Differences that were observed between the oxygen dependent transcriptional profiles at 18 and 34°C are more likely due to stringent selection criteria applied for the determination of regulated genes, than to a real effect of temperature, as most of the genes showing a significant regulation at just one of the two investigated temperatures, showed the same tendency of regulation also at the other temperature. This underlines the hypothesis that *L. weihenstephanensis* is a true environmental species which has no need to adapt to a host.

## Conclusion

This study is the first one that provided a complete genome, a transcriptome and an operon map of a member of the *Listeria* sensu lato phylogenetic clade. The capability of *L. weihenstephanensis* DSM 24698 to reduce nitrate and probably nitrite, which is shared, according to genome comparison, at least partially with all other members of the *Listeria* sensu lato group except for *L. floridensis*, clearly separates this phylogenic group from the members of the *Listeria* sensu stricto group. However, further studies need to be conducted before a reclassification of the *Listeria* sensu lato group in novel genera, which was suggested recently (Orsi and Wiedmann, 2016), should be considered.

## Author contributions

EF: Performed experiments, data analysis, wrote the manuscript. MW: Bioinformatics. CH: Performed together with EF, DNA sequencing, bioinformatics. SS: Supervised the project, discussions, corrected the manuscript. SM: Designed experiments, data analysis, wrote the manuscript.

### Conflict of interest statement

The authors declare that the research was conducted in the absence of any commercial or financial relationships that could be construed as a potential conflict of interest.
