# Supplementary material for: Complete Circular Genome Sequence and Temperature Independent Adaptation to Anaerobiosis of Listeria weihenstephanensis DSM 24698
Source: Front Microbiol. 2017 Sep 1;8:1672. doi: 10.3389/fmicb.2017.01672 (PMC5585140; doi:10.3389/fmicb.2017.01672)
Supplement: Supplementary file 2 [file Table2.DOCX]

**Table S2: List of inferred operons in *L. weihenstephanensis*.**

| Start | Stop | Strand | Number of genes | Genes |
| --- | --- | --- | --- | --- |
| 3635 | 5524 | + | 2 | UE46_00020, UE46_00025 |
| 5648 | 8993 | + | 3 | UE46_00030, UE46_00035, UE46_00040 |
| 12128 | 16423 | + | 6 | UE46_00050, UE46_00055, UE46_00060, UE46_00065, UE46_00070, UE46_00075 |
| 16763 | 20766 | + | 3 | UE46_00080, UE46_00085, UE46_00090 |
| 24543 | 26354 | + | 3 | UE46_00110, UE46_00115, UE46_00120 |
| 26756 | 27394 | - | 2 | UE46_00125, UE46_00130 |
| 37048 | 39951 | - | 2 | UE46_00180, UE46_00185 |
| 43513 | 46990 | + | 4 | UE46_00200, UE46_00205, UE46_00210, UE46_00215 |
| 48640 | 49854 | + | 3 | UE46_00230, UE46_00235, UE46_00240 |
| 50799 | 53051 | - | 2 | UE46_00250, UE46_00255 |
| 62265 | 63755 | + | 2 | UE46_00270, UE46_00275 |
| 68955 | 69351 | + | 2 | UE46_00310, UE46_00315 |
| 69794 | 71026 | + | 3 | UE46_00325, UE46_00330, UE46_00335 |
| 95453 | 97890 | + | 2 | UE46_00420, UE46_00425 |
| 101163 | 103452 | - | 2 | UE46_00440, UE46_00445 |
| 105981 | 110241 | + | 4 | UE46_00455, UE46_00460, UE46_00465, UE46_00470 |
| 115558 | 120738 | + | 4 | UE46_00500, UE46_00505, UE46_00510, UE46_00515 |
| 123452 | 125671 | + | 3 | UE46_00530, UE46_00535, UE46_00540 |
| 125730 | 127715 | + | 4 | UE46_00545, UE46_00550, UE46_00555, UE46_00560 |
| 132034 | 133864 | + | 2 | UE46_00580, UE46_00585 |
| 134313 | 135885 | + | 3 | UE46_00590, UE46_00595, UE46_00600 |
| 136021 | 137281 | + | 2 | UE46_00605, UE46_00610 |
| 137466 | 140794 | + | 2 | UE46_00615, UE46_00620 |
| 141037 | 144573 | - | 3 | UE46_00625, UE46_00630, UE46_00635 |
| 147656 | 148623 | + | 2 | UE46_00660, UE46_00665 |
| 150513 | 152533 | + | 3 | UE46_00675, UE46_00680, UE46_00685 |
| 152575 | 154574 | - | 2 | UE46_00690, UE46_00695 |
| 155263 | 158982 | + | 3 | UE46_00705, UE46_00710, UE46_00715 |
| 161787 | 163121 | + | 2 | UE46_00730, UE46_00735 |
| 171442 | 173821 | + | 3 | UE46_00755, UE46_00760, UE46_00765 |
| 173876 | 175281 | + | 2 | UE46_00770, UE46_00775 |
| 191013 | 194555 | + | 3 | UE46_00820, UE46_00825, UE46_00830 |
| 203918 | 207377 | + | 5 | UE46_00870, UE46_00875, UE46_00880, UE46_00885, UE46_00890 |
| 207477 | 212581 | + | 5 | UE46_00895, UE46_00900, UE46_00905, UE46_00910, UE46_00915 |
| 212851 | 216321 | + | 3 | UE46_00920, UE46_00925, UE46_00930 |
| 220692 | 223918 | + | 3 | UE46_00945, UE46_00950, UE46_00955 |
| 230154 | 230715 | + | 2 | UE46_00990, UE46_00995 |
| 230921 | 231721 | + | 2 | UE46_01000, UE46_01005 |
| 232256 | 232974 | + | 2 | UE46_01010, UE46_01015 |
| 234058 | 234736 | + | 2 | UE46_01025, UE46_01030 |
| 235714 | 236690 | + | 2 | UE46_01040, UE46_01045 |
| 238948 | 240676 | + | 2 | UE46_01065, UE46_01070 |
| 240791 | 244901 | + | 5 | UE46_01075, UE46_01080, UE46_01085, UE46_01090, UE46_01095 |
| 245204 | 249401 | + | 4 | UE46_01100, UE46_01105, UE46_01110, UE46_01115 |
| 250880 | 252569 | + | 2 | UE46_01135, UE46_01140 |
| 254331 | 256329 | + | 2 | UE46_01150, UE46_01155 |
| 265827 | 266850 | + | 3 | UE46_01180, UE46_01185, UE46_01190 |
| 270233 | 270901 | + | 2 | UE46_01220, UE46_01225 |
| 273247 | 278673 | + | 6 | UE46_01240, UE46_01245, UE46_01250, UE46_01255, UE46_01260, UE46_01265 |
| 279514 | 285008 | + | 3 | UE46_01275, UE46_01280, UE46_01285 |
| 285077 | 286212 | + | 2 | UE46_01290, UE46_01295 |
| 286326 | 289043 | + | 2 | UE46_01300, UE46_01305 |
| 290122 | 293019 | + | 4 | UE46_01315, UE46_01320, UE46_01325, UE46_01330 |
| 293399 | 294295 | + | 2 | UE46_01335, UE46_01340 |
| 299623 | 301293 | + | 2 | UE46_01355, UE46_01360 |
| 302830 | 304123 | - | 2 | UE46_01370, UE46_01375 |
| 307252 | 308899 | + | 3 | UE46_01390, UE46_01395, UE46_01400 |
| 323249 | 327789 | + | 4 | UE46_01490, UE46_01495, UE46_01500, UE46_01505 |
| 336050 | 339799 | + | 4 | UE46_01535, UE46_01540, UE46_01545, UE46_01550 |
| 340917 | 344650 | + | 5 | UE46_01560, UE46_01565, UE46_01570, UE46_01575, UE46_01580 |
| 349042 | 349992 | + | 2 | UE46_01615, UE46_01620 |
| 362247 | 364679 | + | 2 | UE46_01650, UE46_01655 |
| 365698 | 368334 | + | 2 | UE46_01665, UE46_01670 |
| 369897 | 371882 | - | 3 | UE46_01680, UE46_01685, UE46_01690 |
| 375361 | 378233 | + | 3 | UE46_01705, UE46_01710, UE46_01715 |
| 378551 | 385003 | + | 14 | UE46_01720, UE46_01725, UE46_01730, UE46_01735, UE46_01740, UE46_01745, UE46_01750, UE46_01755, UE46_01760, UE46_01765, UE46_01770, UE46_01775, UE46_01780, UE46_01785 |
| 385261 | 389847 | + | 8 | UE46_01790, UE46_01795, UE46_01800, UE46_01805, UE46_01810, UE46_01815, UE46_01820, UE46_01825 |
| 390550 | 392941 | + | 5 | UE46_01835, UE46_01840, UE46_01845, UE46_01850, UE46_01855 |
| 397394 | 400262 | + | 3 | UE46_01875, UE46_01880, UE46_01885 |
| 403913 | 408257 | + | 4 | UE46_01905, UE46_01910, UE46_01915, UE46_01920 |
| 409375 | 417303 | + | 7 | UE46_01930, UE46_01935, UE46_01940, UE46_01945, UE46_01950, UE46_01955, UE46_01960 |
| 420630 | 425482 | - | 3 | UE46_01970, UE46_01975, UE46_01980 |
| 427652 | 429613 | + | 3 | UE46_01990, UE46_01995, UE46_02000 |
| 432422 | 436740 | + | 3 | UE46_02020, UE46_02025, UE46_02030 |
| 441520 | 442082 | + | 2 | UE46_02050, UE46_02055 |
| 452801 | 454816 | + | 3 | UE46_02110, UE46_02115, UE46_02120 |
| 455450 | 458298 | + | 3 | UE46_02125, UE46_02130, UE46_02135 |
| 458368 | 461762 | + | 3 | UE46_02140, UE46_02145, UE46_02150 |
| 461964 | 466451 | + | 5 | UE46_02155, UE46_02160, UE46_02165, UE46_02170, UE46_02175 |
| 466653 | 469465 | + | 4 | UE46_02180, UE46_02185, UE46_02190, UE46_02195 |
| 477875 | 481210 | + | 4 | UE46_02230, UE46_02235, UE46_02240, UE46_02245 |
| 482991 | 485218 | + | 3 | UE46_02255, UE46_02260, UE46_02265 |
| 485410 | 487604 | + | 2 | UE46_02270, UE46_02275 |
| 488707 | 490763 | + | 2 | UE46_02290, UE46_02295 |
| 493717 | 495634 | + | 3 | UE46_02310, UE46_02315, UE46_02320 |
| 495875 | 497759 | + | 3 | UE46_02325, UE46_02330, UE46_02335 |
| 511299 | 513120 | - | 2 | UE46_02385, UE46_02390 |
| 513388 | 516602 | + | 3 | UE46_02395, UE46_02400, UE46_02405 |
| 516808 | 519346 | + | 3 | UE46_02410, UE46_02415, UE46_02420 |
| 519650 | 521099 | + | 2 | UE46_02425, UE46_02430 |
| 524552 | 526385 | + | 2 | UE46_02445, UE46_02450 |
| 527272 | 528394 | + | 2 | UE46_02455, UE46_02460 |
| 528500 | 534225 | + | 7 | UE46_02465, UE46_02470, UE46_02475, UE46_02480, UE46_02485, UE46_02490, UE46_02495 |
| 536256 | 538153 | + | 3 | UE46_02510, UE46_02515, UE46_02520 |
| 540193 | 543115 | + | 3 | UE46_02535, UE46_02540, UE46_02545 |
| 544537 | 545715 | - | 2 | UE46_02555, UE46_02560 |
| 546103 | 547668 | + | 2 | UE46_02565, UE46_02570 |
| 547903 | 549922 | + | 2 | UE46_02575, UE46_02580 |
| 553782 | 555644 | + | 2 | UE46_02595, UE46_02600 |
| 556164 | 559089 | + | 3 | UE46_02605, UE46_02610, UE46_02615 |
| 560708 | 563347 | + | 2 | UE46_02625, UE46_02630 |
| 563471 | 565974 | + | 2 | UE46_02635, UE46_02640 |
| 567201 | 569002 | + | 2 | UE46_02650, UE46_02655 |
| 570526 | 571974 | + | 2 | UE46_02665, UE46_02670 |
| 572655 | 575075 | + | 2 | UE46_02680, UE46_02685 |
| 575277 | 577795 | + | 4 | UE46_02690, UE46_02695, UE46_02700, UE46_02705 |
| 578230 | 583080 | + | 2 | UE46_02710, UE46_02715 |
| 583285 | 586452 | + | 4 | UE46_02720, UE46_02725, UE46_02730, UE46_02735 |
| 586649 | 589624 | + | 4 | UE46_02740, UE46_02745, UE46_02750, UE46_02755 |
| 592726 | 594010 | + | 2 | UE46_02775, UE46_02780 |
| 594169 | 594697 | + | 2 | UE46_02785, UE46_02790 |
| 594737 | 595951 | - | 2 | UE46_02795, UE46_02800 |
| 596104 | 598549 | + | 3 | UE46_02805, UE46_02810, UE46_02815 |
| 599950 | 600732 | - | 2 | UE46_02825, UE46_02830 |
| 601162 | 601735 | + | 2 | UE46_02835, UE46_02840 |
| 608715 | 614693 | - | 7 | UE46_02880, UE46_02885, UE46_02890, UE46_02895, UE46_02900, UE46_02905, UE46_02910 |
| 618687 | 621787 | + | 2 | UE46_02930, UE46_02935 |
| 621923 | 623700 | - | 2 | UE46_02940, UE46_02945 |
| 626105 | 628300 | + | 2 | UE46_02965, UE46_02970 |
| 635311 | 637273 | - | 3 | UE46_03070, UE46_03075, UE46_03080 |
| 637564 | 641377 | + | 3 | UE46_03085, UE46_03090, UE46_03095 |
| 643070 | 644807 | + | 2 | UE46_03110, UE46_03115 |
| 647868 | 651734 | + | 4 | UE46_03125, UE46_03130, UE46_03135, UE46_03140 |
| 654139 | 655334 | + | 2 | UE46_03155, UE46_03160 |
| 655565 | 657598 | + | 2 | UE46_03165, UE46_03170 |
| 659315 | 659769 | - | 2 | UE46_03190, UE46_03195 |
| 663306 | 664842 | + | 2 | UE46_03215, UE46_03220 |
| 670269 | 673639 | + | 3 | UE46_03240, UE46_03245, UE46_03250 |
| 673909 | 676197 | + | 2 | UE46_03255, UE46_03260 |
| 679475 | 681172 | + | 3 | UE46_03285, UE46_03290, UE46_03295 |
| 683859 | 687470 | + | 3 | UE46_03315, UE46_03320, UE46_03325 |
| 690685 | 693070 | - | 2 | UE46_03335, UE46_03340 |
| 693240 | 696634 | - | 2 | UE46_03345, UE46_03350 |
| 696987 | 699088 | + | 2 | UE46_03355, UE46_03360 |
| 699817 | 703477 | + | 4 | UE46_03370, UE46_03375, UE46_03380, UE46_03385 |
| 708687 | 710691 | + | 3 | UE46_03420, UE46_03425, UE46_03430 |
| 710772 | 714096 | + | 4 | UE46_03435, UE46_03440, UE46_03445, UE46_03450 |
| 714277 | 716685 | + | 2 | UE46_03455, UE46_03460 |
| 716818 | 717577 | + | 2 | UE46_03465, UE46_03470 |
| 718521 | 720060 | + | 2 | UE46_03480, UE46_03485 |
| 720226 | 723011 | + | 3 | UE46_03490, UE46_03495, UE46_03500 |
| 724212 | 728905 | + | 5 | UE46_03510, UE46_03515, UE46_03520, UE46_03525, UE46_03530 |
| 730073 | 730809 | - | 2 | UE46_03540, UE46_03545 |
| 730915 | 731975 | - | 2 | UE46_03550, UE46_03555 |
| 733583 | 735421 | + | 3 | UE46_03565, UE46_03570, UE46_03575 |
| 735582 | 739621 | + | 4 | UE46_03580, UE46_03585, UE46_03590, UE46_03595 |
| 739663 | 743663 | + | 4 | UE46_03600, UE46_03605, UE46_03610, UE46_03615 |
| 752607 | 754069 | + | 2 | UE46_03660, UE46_03665 |
| 759142 | 762707 | + | 2 | UE46_03680, UE46_03685 |
| 774352 | 775985 | + | 2 | UE46_03725, UE46_03730 |
| 776249 | 779002 | + | 3 | UE46_03735, UE46_03740, UE46_03745 |
| 780827 | 789401 | + | 7 | UE46_03760, UE46_03765, UE46_03770, UE46_03775, UE46_03780, UE46_03785, UE46_03790 |
| 792885 | 795302 | - | 2 | UE46_03810, UE46_03815 |
| 802295 | 804507 | + | 3 | UE46_03865, UE46_03870, UE46_03875 |
| 804539 | 805370 | - | 2 | UE46_03880, UE46_03885 |
| 808564 | 812593 | + | 3 | UE46_03905, UE46_03910, UE46_03915 |
| 823694 | 825552 | - | 3 | UE46_03950, UE46_03955, UE46_03960 |
| 826786 | 828023 | - | 2 | UE46_03970, UE46_03975 |
| 833697 | 834868 | + | 2 | UE46_04015, UE46_04020 |
| 835794 | 836847 | + | 2 | UE46_04025, UE46_04030 |
| 837358 | 838338 | + | 2 | UE46_04040, UE46_04045 |
| 838969 | 839684 | + | 2 | UE46_04055, UE46_04060 |
| 846916 | 847976 | - | 2 | UE46_04085, UE46_04090 |
| 850022 | 853515 | - | 2 | UE46_04105, UE46_04110 |
| 855717 | 857392 | - | 2 | UE46_04125, UE46_04130 |
| 863425 | 865215 | + | 2 | UE46_04140, UE46_04145 |
| 865257 | 868888 | + | 3 | UE46_04150, UE46_04155, UE46_04160 |
| 874293 | 876083 | + | 2 | UE46_04180, UE46_04185 |
| 876136 | 877821 | + | 2 | UE46_04190, UE46_04195 |
| 877974 | 879361 | + | 2 | UE46_04200, UE46_04205 |
| 879848 | 884612 | + | 3 | UE46_04210, UE46_04215, UE46_04220 |
| 888680 | 890764 | + | 3 | UE46_04240, UE46_04245, UE46_04250 |
| 893440 | 894605 | + | 2 | UE46_04265, UE46_04270 |
| 895085 | 896708 | + | 2 | UE46_04280, UE46_04285 |
| 897514 | 899044 | - | 2 | UE46_04295, UE46_04300 |
| 899206 | 900223 | + | 2 | UE46_04305, UE46_04310 |
| 903602 | 905305 | - | 2 | UE46_04330, UE46_04335 |
| 905991 | 907240 | + | 3 | UE46_04345, UE46_04350, UE46_04355 |
| 907777 | 909960 | + | 2 | UE46_04360, UE46_04365 |
| 910620 | 913311 | - | 2 | UE46_04375, UE46_04380 |
| 915073 | 916779 | - | 2 | UE46_04390, UE46_04395 |
| 917352 | 919567 | - | 2 | UE46_04405, UE46_04410 |
| 921693 | 923148 | - | 2 | UE46_04425, UE46_04430 |
| 929384 | 930110 | - | 2 | UE46_04445, UE46_04450 |
| 932380 | 936541 | + | 4 | UE46_04465, UE46_04470, UE46_04475, UE46_04480 |
| 936819 | 941937 | + | 4 | UE46_04485, UE46_04490, UE46_04495, UE46_04500 |
| 944983 | 945665 | - | 2 | UE46_04520, UE46_04525 |
| 947833 | 951921 | - | 4 | UE46_04535, UE46_04540, UE46_04545, UE46_04550 |
| 962992 | 965360 | + | 2 | UE46_04585, UE46_04590 |
| 965925 | 967282 | + | 2 | UE46_04600, UE46_04605 |
| 967447 | 978136 | + | 11 | UE46_04610, UE46_04615, UE46_04620, UE46_04625, UE46_04630, UE46_04635, UE46_04640, UE46_04645, UE46_04650, UE46_04655, UE46_04660 |
| 986854 | 988279 | + | 3 | UE46_04690, UE46_04695, UE46_04700 |
| 989785 | 991866 | + | 2 | UE46_04710, UE46_04715 |
| 992073 | 993526 | - | 2 | UE46_04720, UE46_04725 |
| 1009534 | 1011803 | + | 3 | UE46_04765, UE46_04770, UE46_04775 |
| 1011996 | 1014150 | + | 2 | UE46_04780, UE46_04785 |
| 1014258 | 1018152 | + | 4 | UE46_04790, UE46_04795, UE46_04800, UE46_04805 |
| 1019822 | 1021128 | + | 3 | UE46_04820, UE46_04825, UE46_04830 |
| 1021232 | 1023993 | + | 3 | UE46_04835, UE46_04840, UE46_04845 |
| 1027022 | 1028318 | - | 2 | UE46_04860, UE46_04865 |
| 1028470 | 1029164 | + | 2 | UE46_04870, UE46_04875 |
| 1030089 | 1033285 | - | 2 | UE46_04885, UE46_04890 |
| 1035077 | 1039912 | + | 5 | UE46_04910, UE46_04915, UE46_04920, UE46_04925, UE46_04930 |
| 1040128 | 1040759 | + | 2 | UE46_04935, UE46_04940 |
| 1041074 | 1045967 | + | 8 | UE46_04945, UE46_04950, UE46_04955, UE46_04960, UE46_04965, UE46_04970, UE46_04975, UE46_04980 |
| 1046579 | 1049225 | + | 2 | UE46_04990, UE46_04995 |
| 1049719 | 1052279 | - | 3 | UE46_05010, UE46_05015, UE46_05020 |
| 1063285 | 1064139 | - | 2 | UE46_05070, UE46_05075 |
| 1065043 | 1065697 | + | 2 | UE46_05085, UE46_05090 |
| 1067912 | 1070138 | + | 2 | UE46_05105, UE46_05110 |
| 1070777 | 1071514 | + | 2 | UE46_05115, UE46_05120 |
| 1073228 | 1075021 | - | 3 | UE46_05135, UE46_05140, UE46_05145 |
| 1075293 | 1077333 | + | 7 | UE46_05150, UE46_05155, UE46_05160, UE46_05165, UE46_05170, UE46_05175, UE46_05180 |
| 1077711 | 1084426 | + | 8 | UE46_05190, UE46_05195, UE46_05200, UE46_05205, UE46_05210, UE46_05215, UE46_05220, UE46_05225 |
| 1085568 | 1086937 | + | 5 | UE46_05240, UE46_05245, UE46_05250, UE46_05255, UE46_05260 |
| 1087089 | 1090383 | + | 2 | UE46_05265, UE46_05270 |
| 1090706 | 1093626 | + | 5 | UE46_05275, UE46_05280, UE46_05285, UE46_05290, UE46_05295 |
| 1094198 | 1099407 | + | 5 | UE46_05305, UE46_05310, UE46_05315, UE46_05320, UE46_05325 |
| 1099551 | 1101581 | + | 5 | UE46_05330, UE46_05335, UE46_05340, UE46_05345, UE46_05350 |
| 1101853 | 1103291 | + | 2 | UE46_05355, UE46_05360 |
| 1103471 | 1110883 | + | 5 | UE46_05365, UE46_05370, UE46_05375, UE46_05380, UE46_05385 |
| 1111072 | 1113004 | + | 4 | UE46_05390, UE46_05395, UE46_05400, UE46_05405 |
| 1113837 | 1115880 | - | 2 | UE46_05415, UE46_05420 |
| 1116049 | 1117431 | + | 2 | UE46_05425, UE46_05430 |
| 1117726 | 1119362 | - | 2 | UE46_05435, UE46_05440 |
| 1119901 | 1121336 | - | 2 | UE46_05450, UE46_05455 |
| 1121614 | 1127194 | + | 6 | UE46_05460, UE46_05465, UE46_05470, UE46_05475, UE46_05480, UE46_05485 |
| 1128047 | 1131653 | + | 5 | UE46_05495, UE46_05500, UE46_05505, UE46_05510, UE46_05515 |
| 1134322 | 1136508 | + | 2 | UE46_05530, UE46_05535 |
| 1136692 | 1138199 | + | 2 | UE46_05540, UE46_05545 |
| 1149598 | 1150677 | - | 2 | UE46_05570, UE46_05575 |
| 1150847 | 1152349 | + | 2 | UE46_05580, UE46_05585 |
| 1156826 | 1158025 | - | 2 | UE46_05605, UE46_05610 |
| 1161526 | 1163068 | + | 2 | UE46_05625, UE46_05630 |
| 1163246 | 1165411 | + | 2 | UE46_05635, UE46_05640 |
| 1175585 | 1177730 | + | 2 | UE46_05670, UE46_05675 |
| 1177779 | 1179381 | - | 2 | UE46_05680, UE46_05685 |
| 1181506 | 1183200 | + | 3 | UE46_05705, UE46_05710, UE46_05715 |
| 1183360 | 1186844 | + | 4 | UE46_05720, UE46_05725, UE46_05730, UE46_05735 |
| 1186978 | 1190029 | + | 4 | UE46_05740, UE46_05745, UE46_05750, UE46_05755 |
| 1191475 | 1195433 | + | 4 | UE46_05765, UE46_05770, UE46_05775, UE46_05780 |
| 1202177 | 1203776 | + | 2 | UE46_05825, UE46_05830 |
| 1204494 | 1205729 | + | 2 | UE46_05840, UE46_05845 |
| 1205785 | 1208464 | - | 4 | UE46_05850, UE46_05855, UE46_05860, UE46_05865 |
| 1217104 | 1220082 | + | 3 | UE46_05885, UE46_05890, UE46_05895 |
| 1233972 | 1236688 | + | 2 | UE46_05955, UE46_05960 |
| 1237027 | 1241360 | + | 5 | UE46_05965, UE46_05970, UE46_05975, UE46_05980, UE46_05985 |
| 1242066 | 1243940 | + | 2 | UE46_05995, UE46_06000 |
| 1245274 | 1247834 | + | 3 | UE46_06010, UE46_06015, UE46_06020 |
| 1248267 | 1253719 | - | 4 | UE46_06025, UE46_06030, UE46_06035, UE46_06040 |
| 1259985 | 1260924 | + | 2 | UE46_06070, UE46_06075 |
| 1261859 | 1263600 | + | 2 | UE46_06085, UE46_06090 |
| 1263860 | 1271952 | + | 5 | UE46_06095, UE46_06100, UE46_06105, UE46_06110, UE46_06115 |
| 1274601 | 1277728 | - | 2 | UE46_06130, UE46_06135 |
| 1277982 | 1281417 | + | 6 | UE46_06140, UE46_06145, UE46_06150, UE46_06155, UE46_06160, UE46_06165 |
| 1284328 | 1286717 | + | 3 | UE46_06195, UE46_06200, UE46_06205 |
| 1287788 | 1288999 | - | 2 | UE46_06215, UE46_06220 |
| 1291080 | 1292436 | + | 2 | UE46_06240, UE46_06245 |
| 1292759 | 1296551 | + | 4 | UE46_06250, UE46_06255, UE46_06260, UE46_06265 |
| 1298410 | 1300310 | - | 2 | UE46_06275, UE46_06280 |
| 1301594 | 1303306 | - | 2 | UE46_06290, UE46_06295 |
| 1304985 | 1306183 | + | 2 | UE46_06315, UE46_06320 |
| 1306349 | 1308206 | + | 2 | UE46_06325, UE46_06330 |
| 1309193 | 1312470 | + | 4 | UE46_06340, UE46_06345, UE46_06350, UE46_06355 |
| 1316121 | 1317989 | + | 3 | UE46_06380, UE46_06385, UE46_06390 |
| 1318254 | 1320365 | + | 3 | UE46_06395, UE46_06400, UE46_06405 |
| 1322777 | 1324047 | + | 2 | UE46_06425, UE46_06430 |
| 1328197 | 1332209 | + | 4 | UE46_06450, UE46_06455, UE46_06460, UE46_06465 |
| 1332476 | 1338326 | + | 5 | UE46_06470, UE46_06475, UE46_06480, UE46_06485, UE46_06490 |
| 1338656 | 1342353 | + | 4 | UE46_06495, UE46_06500, UE46_06505, UE46_06510 |
| 1347956 | 1349654 | + | 2 | UE46_06530, UE46_06535 |
| 1351124 | 1356963 | - | 5 | UE46_06550, UE46_06555, UE46_06560, UE46_06565, UE46_06570 |
| 1358249 | 1362145 | - | 3 | UE46_06580, UE46_06585, UE46_06590 |
| 1362777 | 1363756 | + | 2 | UE46_06595, UE46_06600 |
| 1367239 | 1368957 | + | 2 | UE46_06620, UE46_06625 |
| 1369090 | 1370621 | + | 2 | UE46_06630, UE46_06635 |
| 1370774 | 1372647 | - | 2 | UE46_06640, UE46_06645 |
| 1372853 | 1373857 | + | 2 | UE46_06650, UE46_06655 |
| 1375417 | 1377721 | + | 3 | UE46_06665, UE46_06670, UE46_06675 |
| 1378434 | 1380261 | - | 2 | UE46_06685, UE46_06690 |
| 1380447 | 1383496 | + | 3 | UE46_06695, UE46_06700, UE46_06705 |
| 1384271 | 1386021 | + | 2 | UE46_06715, UE46_06720 |
| 1386184 | 1389624 | + | 4 | UE46_06725, UE46_06730, UE46_06735, UE46_06740 |
| 1390824 | 1393142 | + | 2 | UE46_06750, UE46_06755 |
| 1394654 | 1400151 | + | 6 | UE46_06770, UE46_06775, UE46_06780, UE46_06785, UE46_06790, UE46_06795 |
| 1406824 | 1409751 | + | 4 | UE46_06830, UE46_06835, UE46_06840, UE46_06845 |
| 1409927 | 1416051 | + | 6 | UE46_06850, UE46_06855, UE46_06860, UE46_06865, UE46_06870, UE46_06875 |
| 1416171 | 1419133 | + | 3 | UE46_06880, UE46_06885, UE46_06890 |
| 1419766 | 1422429 | + | 3 | UE46_06895, UE46_06900, UE46_06905 |
| 1422458 | 1425544 | - | 3 | UE46_06910, UE46_06915, UE46_06920 |
| 1426843 | 1431351 | - | 4 | UE46_06930, UE46_06935, UE46_06940, UE46_06945 |
| 1433116 | 1436801 | + | 5 | UE46_06960, UE46_06965, UE46_06970, UE46_06975, UE46_06980 |
| 1436986 | 1437945 | + | 2 | UE46_06985, UE46_06990 |
| 1438182 | 1440251 | + | 3 | UE46_06995, UE46_07000, UE46_07005 |
| 1443184 | 1446207 | + | 3 | UE46_07015, UE46_07020, UE46_07025 |
| 1446628 | 1448501 | + | 3 | UE46_07030, UE46_07035, UE46_07040 |
| 1448564 | 1451791 | - | 2 | UE46_07045, UE46_07050 |
| 1452109 | 1453004 | + | 2 | UE46_07055, UE46_07060 |
| 1454028 | 1456712 | + | 2 | UE46_07070, UE46_07075 |
| 1457041 | 1459874 | + | 3 | UE46_07080, UE46_07085, UE46_07090 |
| 1462910 | 1463525 | - | 2 | UE46_07100, UE46_07105 |
| 1466321 | 1469965 | + | 4 | UE46_07120, UE46_07125, UE46_07130, UE46_07135 |
| 1475926 | 1477351 | - | 2 | UE46_07155, UE46_07160 |
| 1479646 | 1485742 | + | 9 | UE46_07175, UE46_07180, UE46_07185, UE46_07190, UE46_07195, UE46_07200, UE46_07205, UE46_07210, UE46_07215 |
| 1490551 | 1493070 | + | 2 | UE46_07225, UE46_07230 |
| 1493199 | 1495398 | + | 3 | UE46_07235, UE46_07240, UE46_07245 |
| 1495576 | 1497495 | + | 2 | UE46_07250, UE46_07255 |
| 1498018 | 1500481 | + | 3 | UE46_07260, UE46_07265, UE46_07270 |
| 1500563 | 1503799 | - | 3 | UE46_07275, UE46_07280, UE46_07285 |
| 1504007 | 1506734 | + | 3 | UE46_07290, UE46_07295, UE46_07300 |
| 1508352 | 1519222 | + | 9 | UE46_07310, UE46_07315, UE46_07320, UE46_07325, UE46_07330, UE46_07335, UE46_07340, UE46_07345, UE46_07350 |
| 1520514 | 1528782 | + | 7 | UE46_07360, UE46_07365, UE46_07370, UE46_07375, UE46_07380, UE46_07385, UE46_07390 |
| 1529211 | 1531370 | - | 2 | UE46_07400, UE46_07405 |
| 1532427 | 1533248 | + | 2 | UE46_07415, UE46_07420 |
| 1539529 | 1540456 | + | 2 | UE46_07450, UE46_07455 |
| 1540736 | 1542608 | + | 2 | UE46_07460, UE46_07465 |
| 1542842 | 1553066 | + | 8 | UE46_07470, UE46_07475, UE46_07480, UE46_07485, UE46_07490, UE46_07495, UE46_07500, UE46_07505 |
| 1553607 | 1555111 | + | 3 | UE46_07515, UE46_07520, UE46_07525 |
| 1556427 | 1558467 | + | 2 | UE46_07535, UE46_07540 |
| 1559881 | 1562812 | + | 2 | UE46_07550, UE46_07555 |
| 1562925 | 1566233 | + | 4 | UE46_07560, UE46_07565, UE46_07570, UE46_07575 |
| 1567093 | 1572368 | + | 3 | UE46_07585, UE46_07590, UE46_07595 |
| 1573420 | 1575123 | + | 2 | UE46_07605, UE46_07610 |
| 1580971 | 1581488 | + | 2 | UE46_07625, UE46_07630 |
| 1582320 | 1584324 | + | 4 | UE46_07640, UE46_07645, UE46_07650, UE46_07655 |
| 1585746 | 1586410 | + | 2 | UE46_07670, UE46_07675 |
| 1586719 | 1587868 | + | 3 | UE46_07680, UE46_07685, UE46_07690 |
| 1588009 | 1588751 | + | 2 | UE46_07695, UE46_07700 |
| 1588897 | 1591009 | + | 2 | UE46_07705, UE46_07710 |
| 1594805 | 1598269 | + | 2 | UE46_07735, UE46_07740 |
| 1598377 | 1602065 | + | 2 | UE46_07745, UE46_07750 |
| 1602187 | 1603868 | + | 2 | UE46_07755, UE46_07760 |
| 1604998 | 1605804 | + | 2 | UE46_07770, UE46_07775 |
| 1605926 | 1610024 | + | 2 | UE46_07780, UE46_07785 |
| 1610564 | 1613589 | + | 2 | UE46_07795, UE46_07800 |
| 1614231 | 1616955 | - | 4 | UE46_07810, UE46_07815, UE46_07820, UE46_07825 |
| 1617159 | 1618150 | + | 2 | UE46_07830, UE46_07835 |
| 1619881 | 1621101 | - | 2 | UE46_07860, UE46_07865 |
| 1621269 | 1622293 | + | 2 | UE46_07870, UE46_07875 |
| 1624894 | 1626965 | - | 2 | UE46_07895, UE46_07900 |
| 1634417 | 1636612 | + | 3 | UE46_07940, UE46_07945, UE46_07950 |
| 1641646 | 1647061 | + | 7 | UE46_07970, UE46_07975, UE46_07980, UE46_07985, UE46_07990, UE46_07995, UE46_08000 |
| 1649910 | 1654860 | + | 3 | UE46_08020, UE46_08025, UE46_08030 |
| 1655014 | 1655989 | + | 2 | UE46_08035, UE46_08040 |
| 1662288 | 1664775 | + | 2 | UE46_08070, UE46_08075 |
| 1667556 | 1668671 | + | 3 | UE46_08095, UE46_08100, UE46_08105 |
| 1673094 | 1677203 | + | 3 | UE46_08125, UE46_08130, UE46_08135 |
| 1679397 | 1683595 | + | 2 | UE46_08155, UE46_08160 |
| 1683727 | 1688581 | + | 4 | UE46_08165, UE46_08170, UE46_08175, UE46_08180 |
| 1688764 | 1693092 | + | 3 | UE46_08185, UE46_08190, UE46_08195 |
| 1700301 | 1702046 | + | 2 | UE46_08220, UE46_08225 |
| 1703058 | 1704839 | + | 2 | UE46_08235, UE46_08240 |
| 1706062 | 1709095 | + | 3 | UE46_08250, UE46_08255, UE46_08260 |
| 1710197 | 1710901 | - | 2 | UE46_08275, UE46_08280 |
| 1711103 | 1713369 | + | 3 | UE46_08285, UE46_08290, UE46_08295 |
| 1719885 | 1725778 | - | 4 | UE46_08325, UE46_08330, UE46_08335, UE46_08340 |
| 1725920 | 1727093 | - | 2 | UE46_08345, UE46_08350 |
| 1732393 | 1734764 | - | 3 | UE46_08375, UE46_08380, UE46_08385 |
| 1734869 | 1737429 | - | 2 | UE46_08390, UE46_08395 |
| 1737716 | 1741243 | - | 3 | UE46_08400, UE46_08405, UE46_08410 |
| 1748468 | 1749923 | - | 2 | UE46_08440, UE46_08445 |
| 1750466 | 1751604 | + | 2 | UE46_08455, UE46_08460 |
| 1751663 | 1753830 | - | 2 | UE46_08465, UE46_08470 |
| 1757484 | 1763319 | - | 5 | UE46_08485, UE46_08490, UE46_08495, UE46_08500, UE46_08505 |
| 1764465 | 1766623 | - | 2 | UE46_08515, UE46_08520 |
| 1766819 | 1769529 | - | 2 | UE46_08525, UE46_08530 |
| 1769797 | 1773130 | - | 4 | UE46_08535, UE46_08540, UE46_08545, UE46_08550 |
| 1773264 | 1775942 | - | 4 | UE46_08555, UE46_08560, UE46_08565, UE46_08570 |
| 1776149 | 1777845 | - | 2 | UE46_08575, UE46_08580 |
| 1778013 | 1779695 | + | 3 | UE46_08585, UE46_08590, UE46_08595 |
| 1780475 | 1784406 | - | 3 | UE46_08605, UE46_08610, UE46_08615 |
| 1784954 | 1791117 | + | 10 | UE46_08620, UE46_08625, UE46_08630, UE46_08635, UE46_08640, UE46_08645, UE46_08650, UE46_08655, UE46_08660, UE46_08665 |
| 1792409 | 1795813 | - | 4 | UE46_08675, UE46_08680, UE46_08685, UE46_08690 |
| 1796226 | 1797093 | + | 2 | UE46_08700, UE46_08705 |
| 1803576 | 1808739 | - | 7 | UE46_08735, UE46_08740, UE46_08745, UE46_08750, UE46_08755, UE46_08760, UE46_08765 |
| 1813571 | 1819360 | - | 5 | UE46_08775, UE46_08780, UE46_08785, UE46_08790, UE46_08795 |
| 1819928 | 1821216 | - | 2 | UE46_08800, UE46_08805 |
| 1827456 | 1830935 | - | 4 | UE46_08830, UE46_08835, UE46_08840, UE46_08845 |
| 1834556 | 1835780 | - | 2 | UE46_08860, UE46_08865 |
| 1836471 | 1841339 | - | 4 | UE46_08870, UE46_08875, UE46_08880, UE46_08885 |
| 1843964 | 1845377 | + | 3 | UE46_08895, UE46_08900, UE46_08905 |
| 1855152 | 1857325 | + | 2 | UE46_08955, UE46_08960 |
| 1858299 | 1860331 | - | 3 | UE46_08970, UE46_08975, UE46_08980 |
| 1861271 | 1865326 | - | 4 | UE46_08990, UE46_08995, UE46_09000, UE46_09005 |
| 1866582 | 1868746 | - | 3 | UE46_09015, UE46_09020, UE46_09025 |
| 1870093 | 1872418 | - | 2 | UE46_09035, UE46_09040 |
| 1872529 | 1876339 | - | 3 | UE46_09045, UE46_09050, UE46_09055 |
| 1878500 | 1883846 | - | 6 | UE46_09070, UE46_09075, UE46_09080, UE46_09085, UE46_09090, UE46_09095 |
| 1884002 | 1884642 | - | 2 | UE46_09100, UE46_09105 |
| 1884858 | 1887751 | - | 3 | UE46_09110, UE46_09115, UE46_09120 |
| 1887893 | 1891436 | - | 3 | UE46_09125, UE46_09130, UE46_09135 |
| 1891573 | 1894621 | - | 2 | UE46_09140, UE46_09145 |
| 1901007 | 1905269 | - | 7 | UE46_09180, UE46_09185, UE46_09190, UE46_09195, UE46_09200, UE46_09205, UE46_09210 |
| 1907296 | 1908700 | - | 2 | UE46_09220, UE46_09225 |
| 1909894 | 1912330 | - | 3 | UE46_09235, UE46_09240, UE46_09245 |
| 1913371 | 1914365 | - | 3 | UE46_09255, UE46_09260, UE46_09265 |
| 1917721 | 1919107 | + | 3 | UE46_09275, UE46_09280, UE46_09285 |
| 1919615 | 1920326 | - | 2 | UE46_09295, UE46_09300 |
| 1922463 | 1924675 | - | 2 | UE46_09320, UE46_09325 |
| 1924852 | 1926989 | + | 2 | UE46_09330, UE46_09335 |
| 1928414 | 1928908 | - | 2 | UE46_09345, UE46_09350 |
| 1929699 | 1933533 | - | 3 | UE46_09360, UE46_09365, UE46_09370 |
| 1933617 | 1935890 | - | 2 | UE46_09375, UE46_09380 |
| 1938870 | 1940456 | + | 2 | UE46_09395, UE46_09400 |
| 1941409 | 1944467 | - | 2 | UE46_09410, UE46_09415 |
| 1946748 | 1949444 | - | 2 | UE46_09425, UE46_09430 |
| 1949891 | 1953201 | - | 3 | UE46_09435, UE46_09440, UE46_09445 |
| 1953720 | 1956340 | - | 2 | UE46_09455, UE46_09460 |
| 1956627 | 1958134 | - | 2 | UE46_09465, UE46_09470 |
| 1958222 | 1960890 | - | 3 | UE46_09475, UE46_09480, UE46_09485 |
| 1963856 | 1966021 | - | 2 | UE46_09505, UE46_09510 |
| 1966707 | 1969057 | - | 2 | UE46_09515, UE46_09520 |
| 1969527 | 1970491 | - | 3 | UE46_09525, UE46_09530, UE46_09535 |
| 1970643 | 1972621 | - | 2 | UE46_09540, UE46_09545 |
| 1972711 | 1974191 | - | 2 | UE46_09550, UE46_09555 |
| 1974371 | 1975758 | - | 2 | UE46_09560, UE46_09565 |
| 1977098 | 1978528 | - | 2 | UE46_09575, UE46_09580 |
| 1978772 | 1982766 | - | 2 | UE46_09585, UE46_09590 |
| 1983092 | 1988985 | - | 6 | UE46_09595, UE46_09600, UE46_09605, UE46_09610, UE46_09615, UE46_09620 |
| 1991421 | 1998367 | - | 6 | UE46_09630, UE46_09635, UE46_09640, UE46_09645, UE46_09650, UE46_09655 |
| 1998527 | 2000931 | - | 2 | UE46_09660, UE46_09665 |
| 2005497 | 2008824 | - | 3 | UE46_09690, UE46_09695, UE46_09700 |
| 2010271 | 2010927 | - | 2 | UE46_09710, UE46_09715 |
| 2011078 | 2012899 | - | 2 | UE46_09720, UE46_09725 |
| 2017038 | 2020554 | - | 2 | UE46_09735, UE46_09740 |
| 2022397 | 2028298 | - | 8 | UE46_09755, UE46_09760, UE46_09765, UE46_09770, UE46_09775, UE46_09780, UE46_09785, UE46_09790 |
| 2030172 | 2033134 | - | 3 | UE46_09805, UE46_09810, UE46_09815 |
| 2037266 | 2039475 | - | 2 | UE46_09845, UE46_09850 |
| 2040322 | 2041876 | - | 2 | UE46_09860, UE46_09865 |
| 2043102 | 2044991 | - | 2 | UE46_09875, UE46_09880 |
| 2051118 | 2056248 | - | 5 | UE46_09910, UE46_09915, UE46_09920, UE46_09925, UE46_09930 |
| 2056826 | 2060991 | - | 3 | UE46_09940, UE46_09945, UE46_09950 |
| 2062567 | 2064280 | - | 2 | UE46_09965, UE46_09970 |
| 2067636 | 2068646 | - | 2 | UE46_09985, UE46_09990 |
| 2074443 | 2076190 | - | 3 | UE46_10010, UE46_10015, UE46_10020 |
| 2082013 | 2083457 | - | 2 | UE46_10065, UE46_10070 |
| 2083945 | 2085860 | - | 2 | UE46_10075, UE46_10080 |
| 2086030 | 2087300 | - | 2 | UE46_10085, UE46_10090 |
| 2091586 | 2093427 | - | 2 | UE46_10120, UE46_10125 |
| 2097156 | 2103451 | - | 7 | UE46_10140, UE46_10145, UE46_10150, UE46_10155, UE46_10160, UE46_10165, UE46_10170 |
| 2105759 | 2108125 | - | 2 | UE46_10185, UE46_10190 |
| 2108920 | 2110933 | - | 2 | UE46_10200, UE46_10205 |
| 2116091 | 2117634 | + | 2 | UE46_10225, UE46_10230 |
| 2118816 | 2121403 | - | 2 | UE46_10240, UE46_10245 |
| 2127637 | 2129259 | - | 3 | UE46_10275, UE46_10280, UE46_10285 |
| 2130759 | 2136956 | - | 5 | UE46_10300, UE46_10305, UE46_10310, UE46_10315, UE46_10320 |
| 2144632 | 2148771 | - | 3 | UE46_10340, UE46_10345, UE46_10350 |
| 2163184 | 2165656 | + | 2 | UE46_10505, UE46_10510 |
| 2168335 | 2170154 | - | 2 | UE46_10530, UE46_10535 |
| 2171671 | 2172710 | - | 2 | UE46_10545, UE46_10550 |
| 2173768 | 2177073 | - | 2 | UE46_10560, UE46_10565 |
| 2177313 | 2180066 | - | 3 | UE46_10570, UE46_10575, UE46_10580 |
| 2195783 | 2197245 | - | 2 | UE46_10665, UE46_10670 |
| 2197581 | 2199236 | - | 2 | UE46_10675, UE46_10680 |
| 2206821 | 2208141 | - | 2 | UE46_10715, UE46_10720 |
| 2220386 | 2222182 | + | 2 | UE46_10750, UE46_10755 |
| 2224333 | 2226415 | - | 5 | UE46_10780, UE46_10785, UE46_10790, UE46_10795, UE46_10800 |
| 2226595 | 2229705 | - | 6 | UE46_10805, UE46_10810, UE46_10815, UE46_10820, UE46_10825, UE46_10830 |
| 2230264 | 2232009 | - | 4 | UE46_10840, UE46_10845, UE46_10850, UE46_10855 |
| 2232788 | 2235045 | - | 5 | UE46_10865, UE46_10870, UE46_10875, UE46_10880, UE46_10885 |
| 2241229 | 2242133 | - | 2 | UE46_10915, UE46_10920 |
| 2252369 | 2254363 | + | 2 | UE46_10935, UE46_10940 |
| 2254532 | 2258899 | - | 4 | UE46_10945, UE46_10950, UE46_10955, UE46_10960 |
| 2259142 | 2263502 | - | 4 | UE46_10965, UE46_10970, UE46_10975, UE46_10980 |
| 2265243 | 2265935 | - | 2 | UE46_11000, UE46_11005 |
| 2266759 | 2268404 | + | 2 | UE46_11015, UE46_11020 |
| 2268791 | 2271053 | - | 2 | UE46_11030, UE46_11035 |
| 2271737 | 2272384 | - | 2 | UE46_11045, UE46_11050 |
| 2275466 | 2276503 | - | 3 | UE46_11070, UE46_11075, UE46_11080 |
| 2276844 | 2278477 | + | 2 | UE46_11085, UE46_11090 |
| 2280446 | 2284670 | - | 7 | UE46_11105, UE46_11110, UE46_11115, UE46_11120, UE46_11125, UE46_11130, UE46_11135 |
| 2287223 | 2287620 | + | 2 | UE46_11150, UE46_11155 |
| 2287875 | 2288807 | - | 2 | UE46_11160, UE46_11165 |
| 2289766 | 2290382 | - | 2 | UE46_11170, UE46_11175 |
| 2297146 | 2300257 | - | 2 | UE46_11205, UE46_11210 |
| 2300506 | 2302154 | - | 2 | UE46_11215, UE46_11220 |
| 2304833 | 2305843 | - | 2 | UE46_11240, UE46_11245 |
| 2309781 | 2311212 | - | 2 | UE46_11265, UE46_11270 |
| 2312058 | 2321739 | - | 4 | UE46_11280, UE46_11285, UE46_11290, UE46_11295 |
| 2327328 | 2334297 | - | 7 | UE46_11320, UE46_11325, UE46_11330, UE46_11335, UE46_11340, UE46_11345, UE46_11350 |
| 2344692 | 2354061 | - | 7 | UE46_11385, UE46_11390, UE46_11395, UE46_11400, UE46_11405, UE46_11410, UE46_11415 |
| 2358370 | 2371092 | - | 12 | UE46_11435, UE46_11440, UE46_11445, UE46_11450, UE46_11455, UE46_11460, UE46_11465, UE46_11470, UE46_11475, UE46_11480, UE46_11485, UE46_11490 |
| 2378794 | 2380579 | - | 2 | UE46_11525, UE46_11530 |
| 2384522 | 2385959 | - | 2 | UE46_11555, UE46_11560 |
| 2386580 | 2388504 | + | 3 | UE46_11570, UE46_11575, UE46_11580 |
| 2389941 | 2390694 | - | 2 | UE46_11595, UE46_11600 |
| 2394960 | 2398185 | - | 2 | UE46_11620, UE46_11625 |
| 2398487 | 2399242 | - | 3 | UE46_11630, UE46_11635, UE46_11640 |
| 2401684 | 2403308 | - | 2 | UE46_11655, UE46_11660 |
| 2403495 | 2405086 | - | 3 | UE46_11665, UE46_11670, UE46_11675 |
| 2407497 | 2410610 | - | 4 | UE46_11690, UE46_11695, UE46_11700, UE46_11705 |
| 2413045 | 2417199 | - | 4 | UE46_11720, UE46_11725, UE46_11730, UE46_11735 |
| 2417558 | 2419289 | - | 2 | UE46_11740, UE46_11745 |
| 2420477 | 2423817 | - | 2 | UE46_11755, UE46_11760 |
| 2423978 | 2427063 | - | 2 | UE46_11765, UE46_11770 |
| 2431673 | 2433509 | - | 2 | UE46_11785, UE46_11790 |
| 2448979 | 2450738 | - | 3 | UE46_11860, UE46_11865, UE46_11870 |
| 2450864 | 2451582 | - | 2 | UE46_11875, UE46_11880 |
| 2451936 | 2454978 | - | 2 | UE46_11885, UE46_11890 |
| 2455234 | 2457329 | - | 2 | UE46_11895, UE46_11900 |
| 2459010 | 2460503 | + | 2 | UE46_11910, UE46_11915 |
| 2460559 | 2467153 | - | 9 | UE46_11920, UE46_11925, UE46_11930, UE46_11935, UE46_11940, UE46_11945, UE46_11950, UE46_11955, UE46_11960 |
| 2467290 | 2473775 | - | 4 | UE46_11965, UE46_11970, UE46_11975, UE46_11980 |
| 2474381 | 2477069 | + | 4 | UE46_11985, UE46_11990, UE46_11995, UE46_12000 |
| 2478734 | 2479355 | - | 2 | UE46_12015, UE46_12020 |
| 2479457 | 2481715 | - | 2 | UE46_12025, UE46_12030 |
| 2482486 | 2484074 | + | 2 | UE46_12035, UE46_12040 |
| 2485553 | 2487439 | + | 2 | UE46_12050, UE46_12055 |
| 2498659 | 2500409 | - | 2 | UE46_12085, UE46_12090 |
| 2500580 | 2503657 | - | 4 | UE46_12095, UE46_12100, UE46_12105, UE46_12110 |
| 2509001 | 2511089 | - | 3 | UE46_12140, UE46_12145, UE46_12150 |
| 2516283 | 2518267 | - | 2 | UE46_12180, UE46_12185 |
| 2523224 | 2527637 | + | 5 | UE46_12215, UE46_12220, UE46_12225, UE46_12230, UE46_12235 |
| 2532139 | 2537613 | - | 5 | UE46_12265, UE46_12270, UE46_12275, UE46_12280, UE46_12285 |
| 2538079 | 2539833 | - | 3 | UE46_12290, UE46_12295, UE46_12300 |
| 2541095 | 2552720 | - | 9 | UE46_12310, UE46_12315, UE46_12320, UE46_12325, UE46_12330, UE46_12335, UE46_12340, UE46_12345, UE46_12350 |
| 2557525 | 2559761 | - | 2 | UE46_12385, UE46_12390 |
| 2559988 | 2565920 | - | 7 | UE46_12395, UE46_12400, UE46_12405, UE46_12410, UE46_12415, UE46_12420, UE46_12425 |
| 2566097 | 2569215 | - | 2 | UE46_12430, UE46_12435 |
| 2569334 | 2572845 | - | 3 | UE46_12440, UE46_12445, UE46_12450 |
| 2580801 | 2582296 | - | 2 | UE46_12510, UE46_12515 |
| 2583653 | 2587142 | - | 4 | UE46_12525, UE46_12530, UE46_12535, UE46_12540 |
| 2589826 | 2592414 | + | 2 | UE46_12555, UE46_12560 |
| 2594513 | 2595846 | - | 2 | UE46_12575, UE46_12580 |
| 2596035 | 2599844 | - | 3 | UE46_12585, UE46_12590, UE46_12595 |
| 2604217 | 2606168 | - | 2 | UE46_12625, UE46_12630 |
| 2606432 | 2607303 | + | 2 | UE46_12635, UE46_12640 |
| 2623852 | 2625438 | - | 3 | UE46_12700, UE46_12705, UE46_12710 |
| 2632257 | 2634074 | - | 2 | UE46_12730, UE46_12735 |
| 2635867 | 2642441 | - | 8 | UE46_12750, UE46_12755, UE46_12760, UE46_12765, UE46_12770, UE46_12775, UE46_12780, UE46_12785 |
| 2645670 | 2648532 | - | 3 | UE46_12800, UE46_12805, UE46_12810 |
| 2653075 | 2657239 | - | 5 | UE46_12840, UE46_12845, UE46_12850, UE46_12855, UE46_12860 |
| 2662113 | 2668587 | + | 4 | UE46_12880, UE46_12885, UE46_12890, UE46_12895 |
| 2673632 | 2675037 | - | 3 | UE46_12905, UE46_12910, UE46_12915 |
| 2685730 | 2686810 | - | 2 | UE46_12960, UE46_12965 |
| 2694766 | 2698775 | - | 4 | UE46_13005, UE46_13010, UE46_13015, UE46_13020 |
| 2705071 | 2705935 | - | 2 | UE46_13045, UE46_13050 |
| 2717286 | 2720388 | + | 2 | UE46_13100, UE46_13105 |
| 2722719 | 2726139 | - | 3 | UE46_13120, UE46_13125, UE46_13130 |
| 2726906 | 2728862 | - | 2 | UE46_13140, UE46_13145 |
| 2729024 | 2729830 | + | 2 | UE46_13150, UE46_13155 |
| 2730063 | 2730805 | + | 2 | UE46_13160, UE46_13165 |
| 2734607 | 2744973 | - | 9 | UE46_13185, UE46_13190, UE46_13195, UE46_13200, UE46_13205, UE46_13210, UE46_13215, UE46_13220, UE46_13225 |
| 2745269 | 2746431 | + | 2 | UE46_13230, UE46_13235 |
| 2746542 | 2751614 | - | 3 | UE46_13240, UE46_13245, UE46_13250 |
| 2757887 | 2759290 | + | 2 | UE46_13280, UE46_13285 |
| 2761104 | 2763070 | - | 3 | UE46_13295, UE46_13300, UE46_13305 |
| 2763535 | 2765169 | + | 2 | UE46_13310, UE46_13315 |
| 2767805 | 2771194 | + | 3 | UE46_13335, UE46_13340, UE46_13345 |
| 2771276 | 2773657 | + | 3 | UE46_13350, UE46_13355, UE46_13360 |
| 2774018 | 2774999 | - | 2 | UE46_13365, UE46_13370 |
| 2779735 | 2780857 | - | 2 | UE46_13400, UE46_13405 |
| 2782261 | 2791651 | - | 5 | UE46_13415, UE46_13420, UE46_13425, UE46_13430, UE46_13435 |
| 2793481 | 2797035 | - | 3 | UE46_13455, UE46_13460, UE46_13465 |
| 2797871 | 2803497 | - | 5 | UE46_13470, UE46_13475, UE46_13480, UE46_13485, UE46_13490 |
| 2807653 | 2809195 | - | 2 | UE46_13505, UE46_13510 |
| 2811557 | 2816743 | - | 5 | UE46_13525, UE46_13530, UE46_13535, UE46_13540, UE46_13545 |
| 2820977 | 2822427 | - | 4 | UE46_13565, UE46_13570, UE46_13575, UE46_13580 |
| 2823519 | 2824029 | - | 2 | UE46_13585, UE46_13590 |
| 2829688 | 2837166 | + | 10 | UE46_13610, UE46_13615, UE46_13620, UE46_13625, UE46_13630, UE46_13635, UE46_13640, UE46_13645, UE46_13650, UE46_13655 |
| 2838504 | 2839856 | - | 3 | UE46_13665, UE46_13670, UE46_13675 |
| 2843443 | 2850769 | - | 7 | UE46_13690, UE46_13695, UE46_13700, UE46_13705, UE46_13710, UE46_13715, UE46_13720 |
| 2852246 | 2854114 | - | 3 | UE46_13730, UE46_13735, UE46_13740 |
| 2865181 | 2866613 | - | 3 | UE46_13775, UE46_13780, UE46_13785 |
| 2866726 | 2869482 | - | 2 | UE46_13790, UE46_13795 |
| 2869644 | 2874542 | - | 6 | UE46_13800, UE46_13805, UE46_13810, UE46_13815, UE46_13820, UE46_13825 |
| 2874586 | 2881355 | - | 6 | UE46_13830, UE46_13835, UE46_13840, UE46_13845, UE46_13850, UE46_13855 |
| 2901365 | 2902216 | - | 2 | UE46_13935, UE46_13940 |
| 2902583 | 2905820 | - | 4 | UE46_13945, UE46_13950, UE46_13955, UE46_13960 |
| 2915305 | 2920091 | - | 5 | UE46_14020, UE46_14025, UE46_14030, UE46_14035, UE46_14040 |
| 2920132 | 2922823 | - | 2 | UE46_14045, UE46_14050 |
| 2926589 | 2929220 | - | 3 | UE46_14070, UE46_14075, UE46_14080 |
| 2931046 | 2932395 | - | 2 | UE46_14095, UE46_14100 |
| 2941735 | 2942573 | - | 2 | UE46_14145, UE46_14150 |
| 2943165 | 2944802 | - | 2 | UE46_14155, UE46_14160 |
| 2953198 | 2956601 | - | 3 | UE46_14190, UE46_14195, UE46_14200 |
| 2956814 | 2958467 | + | 2 | UE46_14205, UE46_14210 |
| 2960224 | 2962358 | + | 2 | UE46_14220, UE46_14225 |
| 2962971 | 2963789 | + | 2 | UE46_14235, UE46_14240 |
| 2963816 | 2966301 | - | 2 | UE46_14245, UE46_14250 |
| 2967857 | 2971557 | - | 3 | UE46_14260, UE46_14265, UE46_14270 |
| 2977188 | 2984552 | - | 10 | UE46_14300, UE46_14305, UE46_14310, UE46_14315, UE46_14320, UE46_14325, UE46_14330, UE46_14335, UE46_14340, UE46_14345 |
| 2990291 | 2992484 | + | 2 | UE46_14375, UE46_14380 |
| 2996200 | 2996849 | - | 2 | UE46_14400, UE46_14405 |
| 3000637 | 3003048 | + | 2 | UE46_14425, UE46_14430 |
| 3011723 | 3013081 | - | 2 | UE46_14470, UE46_14475 |
| 3013641 | 3014695 | + | 2 | UE46_14480, UE46_14485 |
| 3017757 | 3020671 | - | 2 | UE46_14500, UE46_14505 |
| 3020728 | 3023675 | - | 3 | UE46_14510, UE46_14515, UE46_14520 |
| 3032111 | 3035293 | - | 2 | UE46_14545, UE46_14550 |
| 3035618 | 3038667 | + | 3 | UE46_14555, UE46_14560, UE46_14565 |
| 3040482 | 3041227 | - | 2 | UE46_14575, UE46_14580 |
| 3044551 | 3049212 | - | 4 | UE46_14600, UE46_14605, UE46_14610, UE46_14615 |
| 3053883 | 3056471 | - | 3 | UE46_14640, UE46_14645, UE46_14650 |
| 3057702 | 3059320 | - | 2 | UE46_14660, UE46_14665 |
| 3059974 | 3061423 | - | 2 | UE46_14675, UE46_14680 |
| 3068849 | 3073579 | - | 7 | UE46_14715, UE46_14720, UE46_14725, UE46_14730, UE46_14735, UE46_14740, UE46_14745 |
| 3076846 | 3077548 | + | 2 | UE46_14760, UE46_14765 |
| 3087744 | 3089788 | - | 2 | UE46_14795, UE46_14800 |
| 3089896 | 3091871 | + | 3 | UE46_14805, UE46_14810, UE46_14815 |
| 3093774 | 3094648 | - | 2 | UE46_14830, UE46_14835 |
| 3094908 | 3096989 | - | 2 | UE46_14840, UE46_14845 |
| 3099866 | 3106343 | - | 5 | UE46_14870, UE46_14875, UE46_14880, UE46_14885, UE46_14890 |
| 3109302 | 3111249 | - | 2 | UE46_14910, UE46_14915 |
| 3115163 | 3117110 | + | 2 | UE46_14925, UE46_14930 |
| 3117159 | 3120424 | - | 4 | UE46_14935, UE46_14940, UE46_14945, UE46_14950 |
| 3125404 | 3131297 | - | 4 | UE46_14960, UE46_14965, UE46_14970, UE46_14975 |
| 3136415 | 3136984 | - | 2 | UE46_15005, UE46_15010 |
| 3139105 | 3140772 | - | 2 | UE46_15020, UE46_15025 |
| 3142294 | 3147316 | - | 4 | UE46_15035, UE46_15040, UE46_15045, UE46_15050 |
| 3156325 | 3159129 | - | 2 | UE46_15085, UE46_15090 |
| 3159268 | 3161067 | - | 2 | UE46_15095, UE46_15100 |
| 3162161 | 3165174 | - | 3 | UE46_15110, UE46_15115, UE46_15120 |
| 3166574 | 3173511 | - | 6 | UE46_15135, UE46_15140, UE46_15145, UE46_15150, UE46_15155, UE46_15160 |
| 3173847 | 3178574 | - | 4 | UE46_15165, UE46_15170, UE46_15175, UE46_15180 |
| 3179886 | 3180455 | - | 2 | UE46_15190, UE46_15195 |
| 3188660 | 3192755 | + | 2 | UE46_15255, UE46_15260 |
| 3192792 | 3195692 | - | 4 | UE46_15265, UE46_15270, UE46_15275, UE46_15280 |
| 3197672 | 3200797 | - | 3 | UE46_15295, UE46_15300, UE46_15305 |
| 3205116 | 3206381 | + | 2 | UE46_15325, UE46_15330 |
| 3206977 | 3208784 | - | 2 | UE46_15340, UE46_15345 |
| 3212339 | 3214494 | - | 3 | UE46_15360, UE46_15365, UE46_15370 |
| 3218453 | 3220092 | - | 2 | UE46_15390, UE46_15395 |
| 3224312 | 3226775 | - | 4 | UE46_15415, UE46_15420, UE46_15425, UE46_15430 |
| 3239605 | 3241525 | - | 2 | UE46_15495, UE46_15500 |
| 3248319 | 3251517 | - | 2 | UE46_15530, UE46_15535 |
| 3252290 | 3256284 | - | 2 | UE46_15545, UE46_15550 |
| 3259506 | 3261933 | - | 2 | UE46_15570, UE46_15575 |
| 3279972 | 3281172 | - | 3 | UE46_15630, UE46_15635, UE46_15640 |
| 3289002 | 3289501 | - | 2 | UE46_15670, UE46_15675 |
| 3297197 | 3299292 | + | 2 | UE46_15685, UE46_15690 |
| 3301859 | 3304490 | - | 4 | UE46_15710, UE46_15715, UE46_15720, UE46_15725 |
| 3313123 | 3314942 | - | 2 | UE46_15750, UE46_15755 |
| 3316393 | 3322085 | - | 7 | UE46_15765, UE46_15770, UE46_15775, UE46_15780, UE46_15785, UE46_15790, UE46_15795 |
| 3329516 | 3330537 | - | 2 | UE46_15815, UE46_15820 |
| 3340399 | 3343213 | - | 2 | UE46_15860, UE46_15865 |
| 3346271 | 3351441 | - | 7 | UE46_15880, UE46_15885, UE46_15890, UE46_15895, UE46_15900, UE46_15905, UE46_15910 |
| 3362669 | 3365455 | - | 3 | UE46_15950, UE46_15955, UE46_15960 |
| 3365513 | 3369080 | - | 3 | UE46_15965, UE46_15970, UE46_15975 |
| 3370303 | 3374331 | - | 4 | UE46_15985, UE46_15990, UE46_15995, UE46_16000 |
| 3375823 | 3377464 | + | 2 | UE46_16010, UE46_16015 |
| 3382863 | 3385288 | - | 3 | UE46_16045, UE46_16050, UE46_16055 |
| 3385414 | 3386996 | - | 2 | UE46_16060, UE46_16065 |
| 3393840 | 3397417 | - | 3 | UE46_16090, UE46_16095, UE46_16100 |
| 3397745 | 3399327 | - | 2 | UE46_16105, UE46_16110 |
| 3400361 | 3404183 | + | 2 | UE46_16120, UE46_16125 |
| 3404242 | 3406118 | - | 3 | UE46_16130, UE46_16135, UE46_16140 |
