# Supplementary material for: Complete Circular Genome Sequence and Temperature Independent Adaptation to Anaerobiosis of Listeria weihenstephanensis DSM 24698
Source: Front Microbiol. 2017 Sep 1;8:1672. doi: 10.3389/fmicb.2017.01672 (PMC5585140; doi:10.3389/fmicb.2017.01672)
Supplement: Supplementary file 5 [file Table5.DOCX]

**Table S5: Semiquantitative determination of nitrite in a *L. weihenstephanensis* DSM 24698** **anaerobic** **culture.**

| **experiment 1** | | | **experiment 2** | | | **experiment 3** | | |
| --- | --- | --- | --- | --- | --- | --- | --- | --- |
| set-up:  modified minimal medium  **0.1% glucose**  0 or 10 mM NaNO_3_ | | | set-up:  modified minimal medium  **0.5% glucose**  0 or 10 mM NaNO_3_ | | | set-up:  modified minimal medium  **1.0% glucose**  0 or 10 mM NaNO_3_ | | |
| time point  (h) | mg/l nitrite  (0 mM NaNO_3_) | mg/l nitrite  (10 mM NaNO_3_) | time point  (h) | mg/l nitrite  (0 mM NaNO_3_) | mg/l nitrite  (10 mM NaNO_3_) | time point  (h) | mg/l nitrite  (0 mM NaNO_3_) | mg/l nitrite  (10 mM NaNO_3_) |
| 0 | 0 | 0 | 0 | 0 | 0 | 0 | 0 | 0 |
| 2 | 0 | 0 | 2 | 0 | 0 | 2 | 0 | 0 |
| 3 | 0 | 0 | 3 | 0 | 0 | 3 | 0 | 0 |
| 4 | 0 | 2 | 4 | 0 | 0 | 4 | 0 | 0 |
| 5 | 0 | 20-50 | 5 | 0 | 0 | 5 | 0 | 0 |
| 6.5 | 0 | 50 | 6.5 | 0 | 20 | 6.5 | 0 | 0 |
| 7.5 | 0 | 50 | 7.5 | 0 | 20 | 7.5 | 0 | 0 |
| 8.5 | 0 | 100 | 8.5 | 0 | 20 | 8.5 | 0 | 0-20 |
| 9.5 | 0 | 100 | 9.5 | 0 | 20 | 9.5 | 0 | 0-20 |
| 10.5 | 0 | 200 | 10.5 | 0 | 50 | 10.5 | 0 | 20 |
| 11.5 | 0 | 200 | 11.5 | 0 | 50 | 11.5 | 0 | 20 |
| 24.5 | 0 | 500 | 24.5 | 0 | 200 | 24.5 | 0 | 100 |

10 mM nitrite would correspond to 460 mg/l.

The nitrite concentration was determined with colorimetric test strips (MERCKOQUANT® test strips for nitrite with a measuring range of 2 to 80 mg/l NO2- and of 0.1 to 3 g/l NO2- from Merck, Darmstadt, Germany),
