# Supplementary material for: Complete Circular Genome Sequence and Temperature Independent Adaptation to Anaerobiosis of Listeria weihenstephanensis DSM 24698
Source: Front Microbiol. 2017 Sep 1;8:1672. doi: 10.3389/fmicb.2017.01672 (PMC5585140; doi:10.3389/fmicb.2017.01672)
Supplement: Supplementary file 6 [file DataSheet1.DOCX]

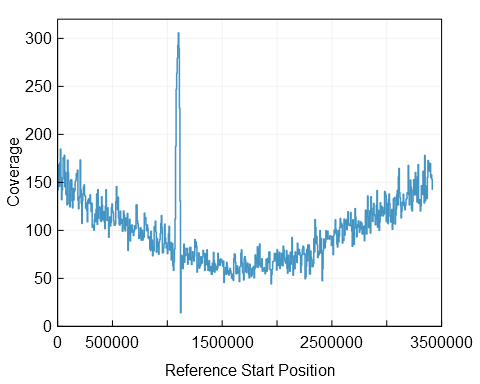
Fig. S1

Fig. S1: Coverage blot of the *L. weihenstephansis* genome sequencing. Reads were mapped against the assembled sequence starting from the origin of replication.
